# Supplementary figures and images for: LIPL-1 and LIPL-2 are TCER-1-regulated lysosomal lipases with distinct roles in immunity and fertility
Source: PLoS Genet. 2025 Dec 12;21(12):e1011804. doi: 10.1371/journal.pgen.1011804 (PMC12716718; doi:10.1371/journal.pgen.1011804)

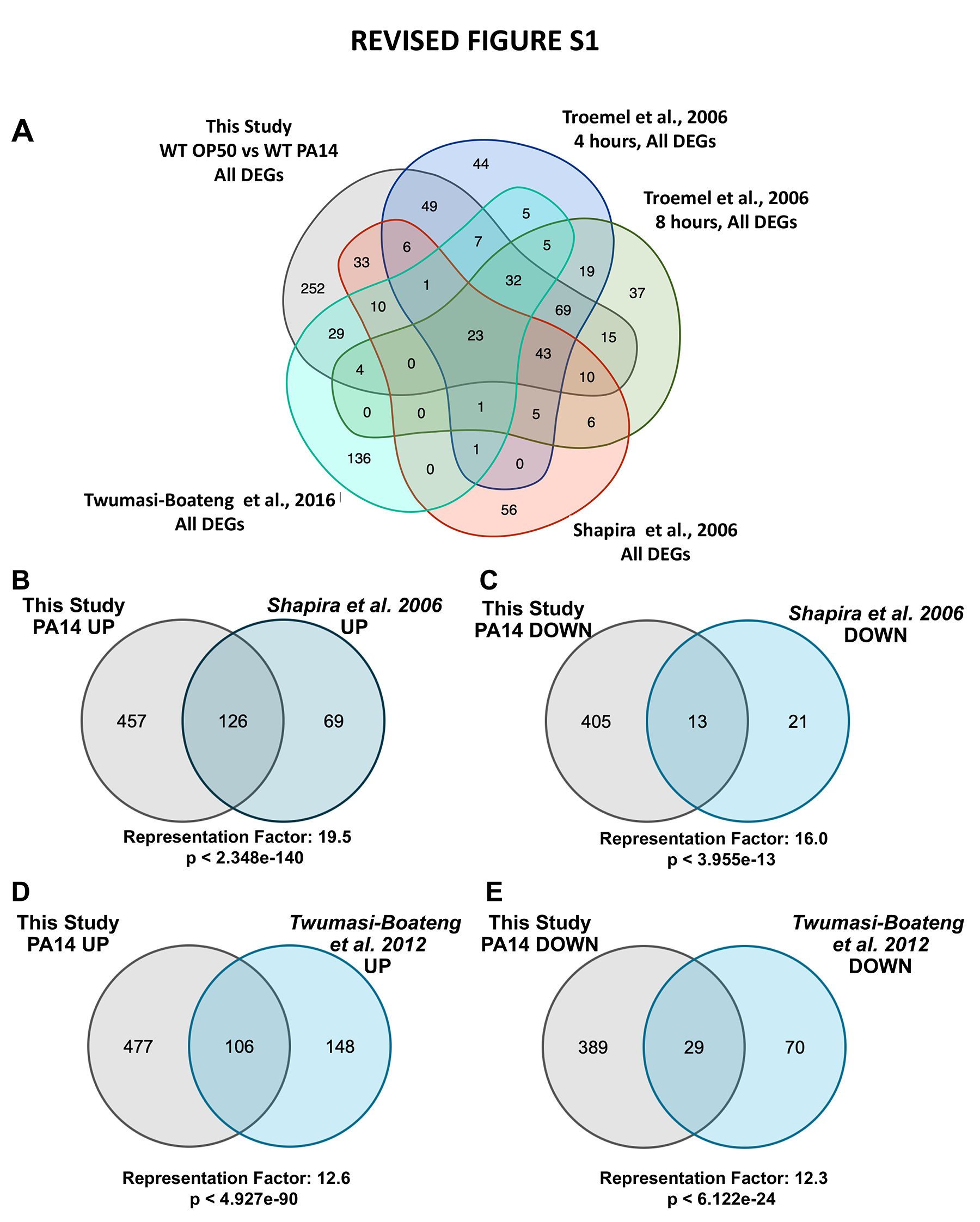

Supplement: S1 Fig — A: All genes up- and down-regulated upon WT animals’ exposure to PA14 for 8 hours in this study compared with genes identified by Troemel et al. after 4 hours and 8 hours of exposure [27]. B-E: Comparisons of DEGs upregulated (B, D) or downregulated (C, E) on PA14 with genes identified by Shapira et al., 2006 (B, C) [26] and Twumasi-Boateng et al. 2012 (D, E) [47]. RF: Representation Factor. Statistical significance of overlap between gene sets calculated using hypergeometric probability formula with normal approximation (see Methods). (TIF) [file pgen.1011804.s001.tif]

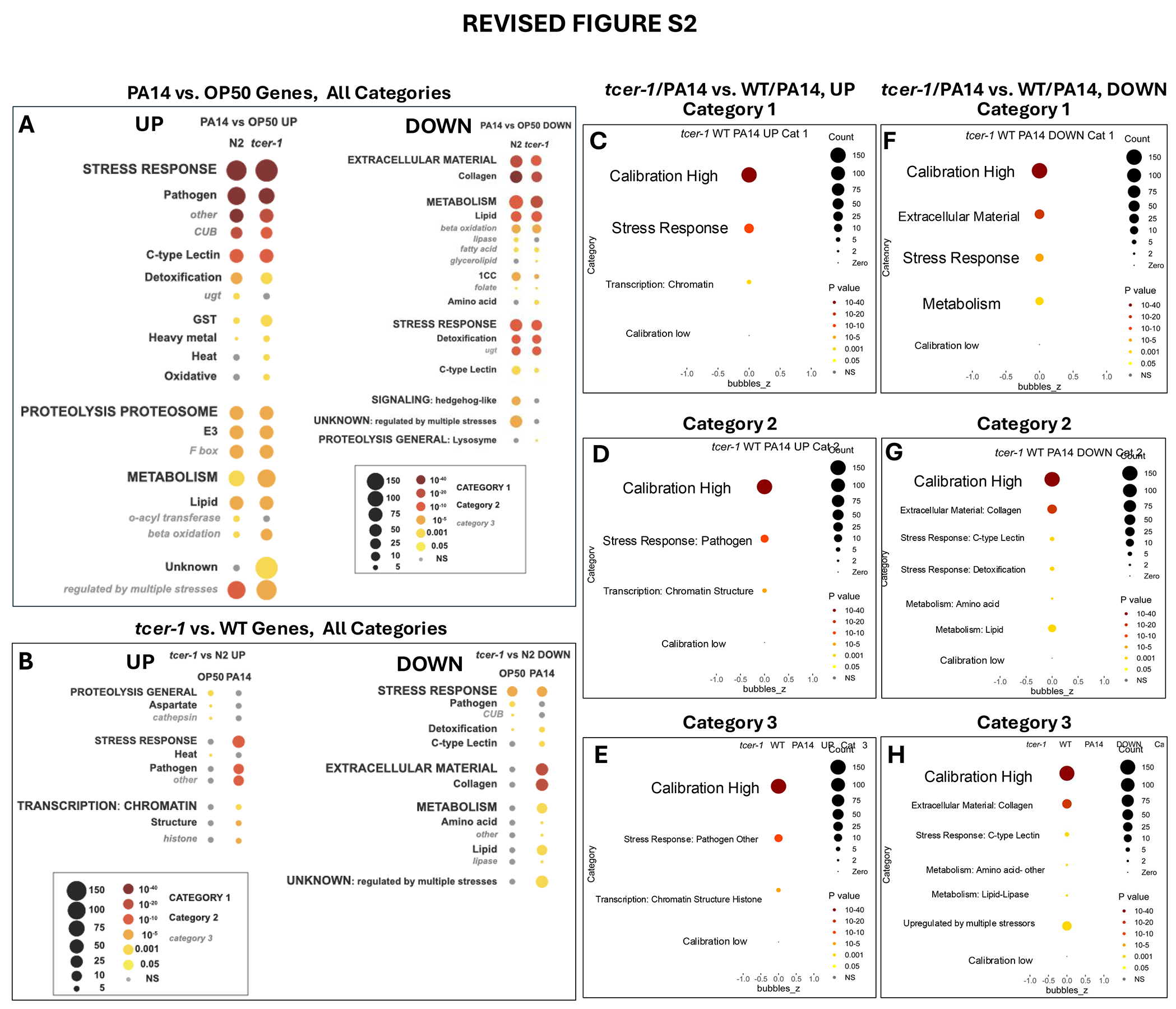

Supplement: S2 Fig — Analysis of gene expression changes in WT animals and tcer-1 mutants upon PA14 infection. Panels A and B show the global transcriptional response driven by infection (A) and genotype (B), while panels C-H detail category-specific changes in tcer-1 mutants on PA14 compared to WT animals on PA14. A: Bubble plot of genes upregulated (UP) or downregulated (right) in WT (N2) and tcer-1 mutants following PA14 exposure compared to OP50. Results of Categories 1, 2 and 3 are shown. B: Comparison of genes up- or downregulated in tcer-1 mutants relative to WT on OP50 or on PA14 infection. Upregulated genes (UP) and downregulated genes (right) are plotted across three categories as in A. C-H: Detailed breakdown of genes upregulated (C-E) or downregulated (F-H) in tcer-1 mutants versus WT under PA14 infection across three hierarchical categories, 1 (C, F), 2 (D, G) and 3 (E, H). Bubble size represents the number of genes in each WormCat category, and color intensity reflects statistical significance. (TIF) [file pgen.1011804.s002.tif]

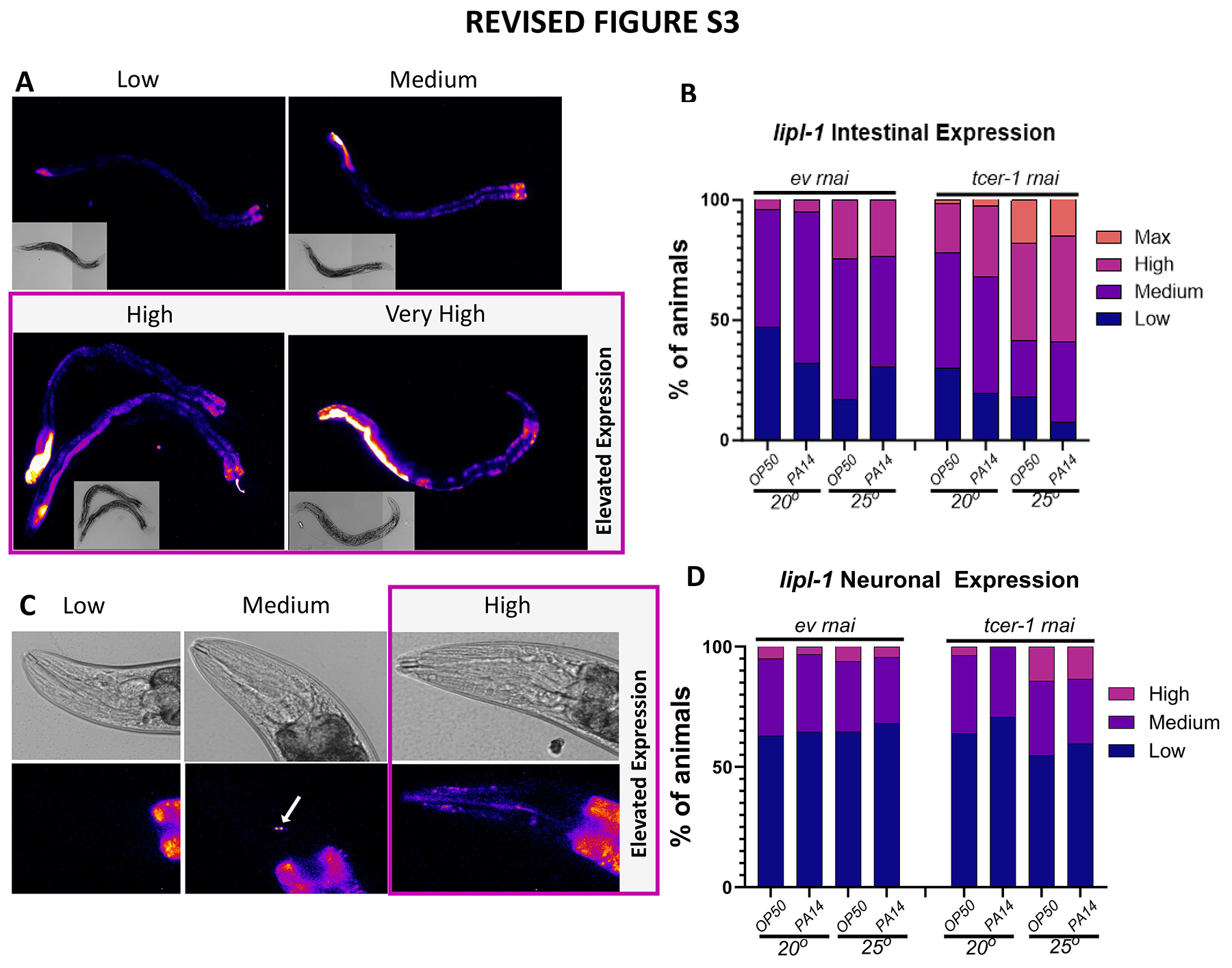

Supplement: S3 Fig — Fluorescence levels quantified based on area and observable intensity variation (see Methods). A, B: Intestinal Expression. A: Representative images of each category pseudocolored with ImageJ LUT Fire. Low- dim fluorescence primarily visible in posterior and anterior intestine. No areas of high intensity; posterior fluorescence limited to region near tail. Medium- posterior fluorescence between vulva and tail. Some areas of intermediate intensity in anterior intestine. High- posterior fluorescence uniformly extended up to the vulva or with multiple areas of bright intensity. Very High– posterior intestine with very bright fluorescence that extends into anterior half beyond the vulva and intermediate fluorescence extended at least to the vulva. Purple boxes indicate categories quantified in Fig 2E, 2F. B: Quantification of percent of population in each category. Data from 3 pooled biological replicates. EV: Empty vector control. (ev RNAi till L4 then OP50, 20°C, n = 81), (ev RNAi till L4 then PA14 20°C, n = 62), (tcer-1 RNAi till L4 then OP50, 20°C, n = 83), (tcer-1 RNAi till L4 then PA14 20°C, n = 41), (ev RNAi till L4 then OP50, 25°C, n = 82), (ev RNAi till L4 then PA14, 25°C, n = 69), (tcer-1 RNAi till L4 then OP50, 25°C, n = 84), (tcer-1 RNAi till L4 then PA14, 25°C, n = 68) C, D: Expression in Head Region. C: Representative images of each category pseudocolored with ImageJ LUT Fire. Low- No fluorescence visible. Medium- 1 or 2 puncta seen. High- More than 2 puncta, or diffuse, non-punctate expression. D: Quantification of data from 3 pooled biological replicates. (ev RNAi till L4 then OP50 20°C, n = 81), (ev RNAi till L4 then PA14 20°C, n = 62), (tcer-1 RNAi till L4 then OP50 20°C, n = 83), (tcer-1 RNAi till L4 then PA14 20°C, n = 41), (ev RNAi till L4 then OP50 25°C, n = 82), (ev RNAi till L4 then PA14 25°C, n = 69), (tcer-1 RNAi OP50 25°C, n = 84), (tcer-1 RNAi PA14 25°C, n = 67). (TIF) [file pgen.1011804.s003.tif]

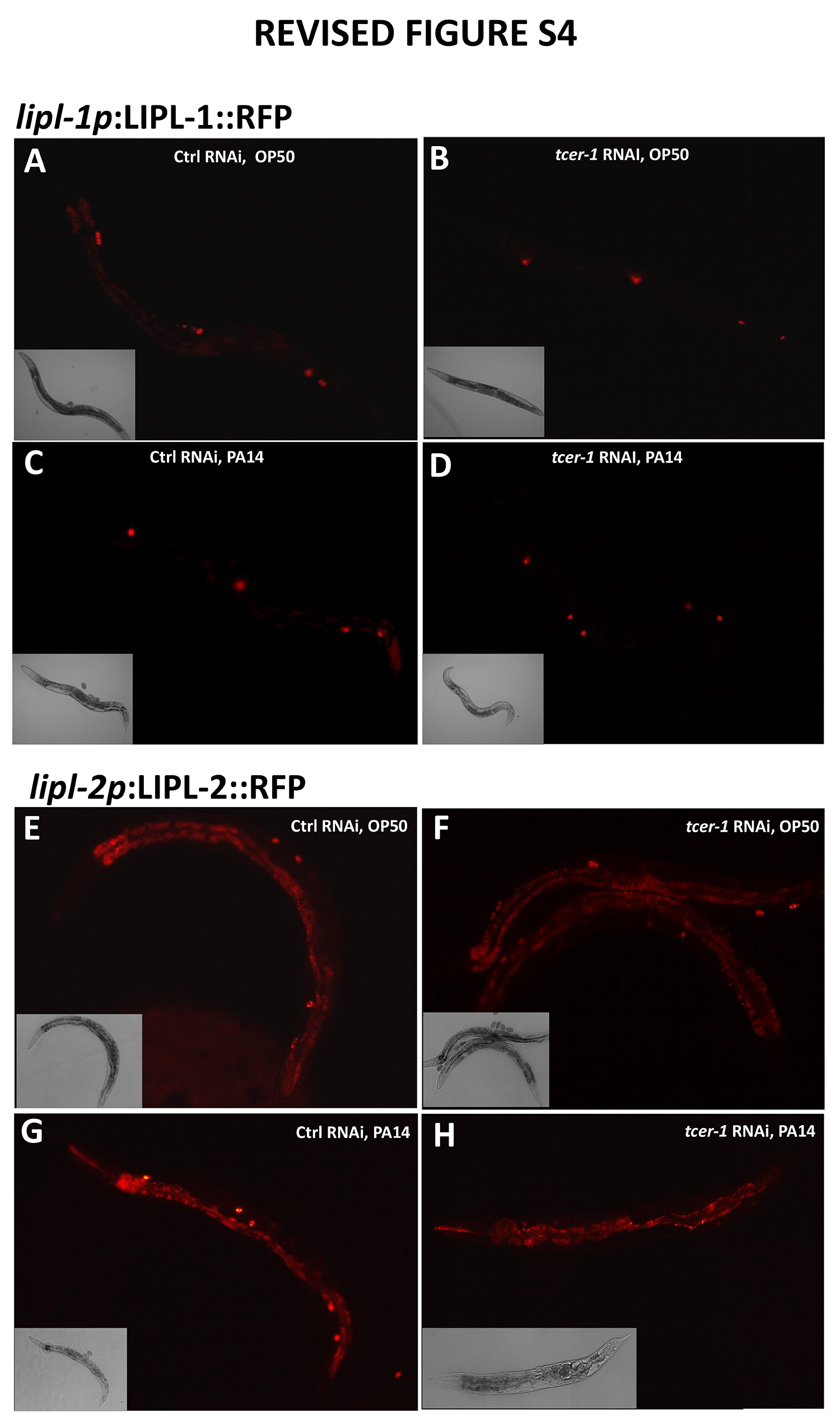

Supplement: S4 Fig — A-D and E-H show representative images of Day 1 adults with lipl-1p::LIPL-1::RFP and lipl-2p::LIPL-2::RFP expression, respectively, that was predominantly seen in coelomocytes. Animals were raised on bacteria expressing control empty vector (Ctrl) or tcer-1 dsRNA (RNAi) until young adulthood, transferred to plates seeded with PA14 or OP50 bacteria and incubated for 8 hours at 25 °C. Images pseudocolored with ImageJ LUT RedHOT showing very low expression predominantly in coelomocytes (with high intestinal autofluorescence in E-H). (TIF) [file pgen.1011804.s004.tif]

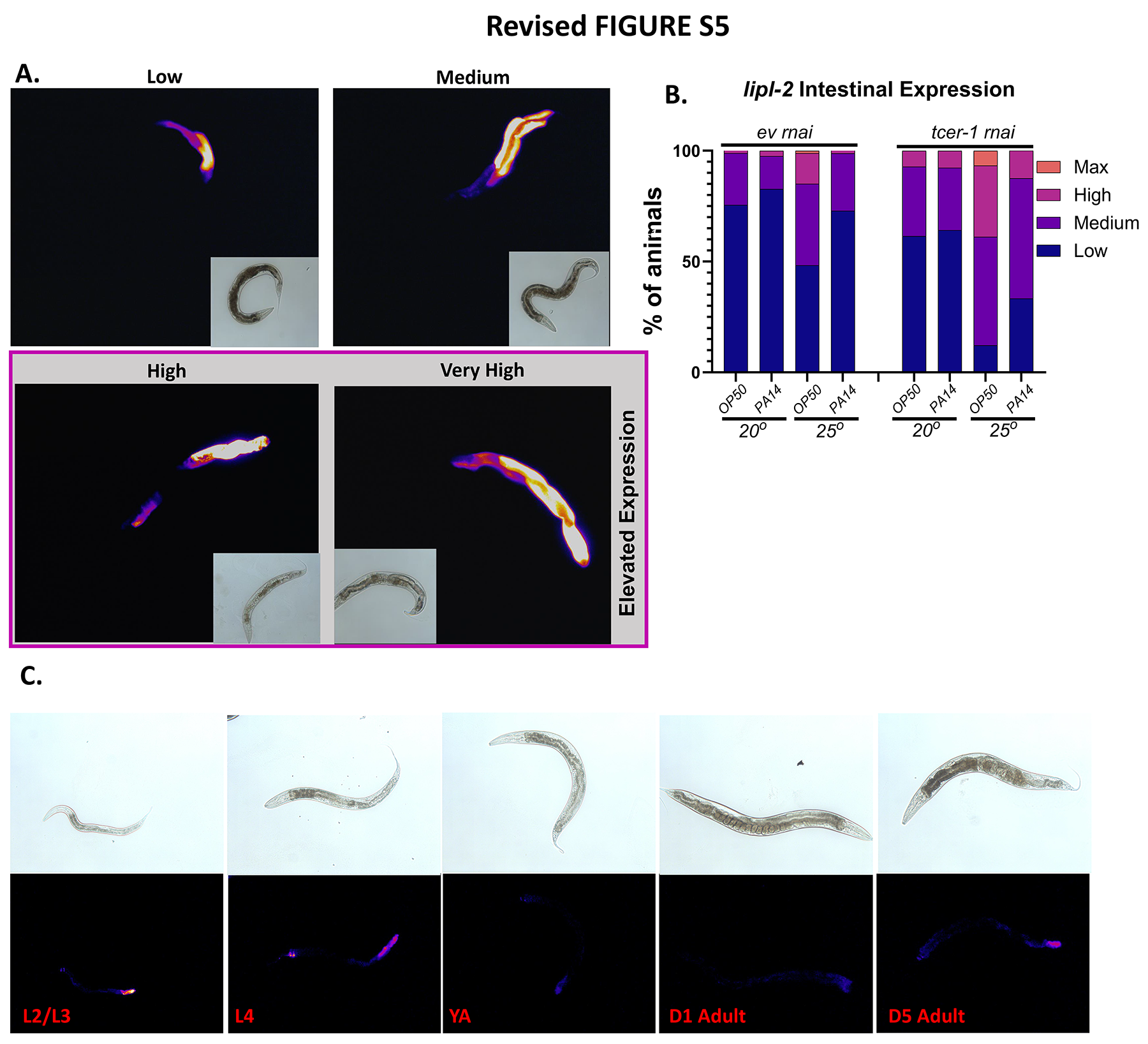

Supplement: S5 Fig — Expression levels in a population were variable and primarily observed in intestines. Fluorescence quantified based on area and observable intensity variation (see Methods). A, B: Intestinal Expression. A: Representative images of each category pseudocolored with ImageJ LUT Fire. Low- dim fluorescence primarily visible in posterior intestine limited to region between vulva and tail. Brighter fluorescence, if present, was restricted to tail. Medium- medium fluorescence in region between vulva and tail. Some area of increased intensity which extended past tail into anterior regions. High – broader posterior signal extending beyond vulva to anterior intestine with areas of bright intensity in posterior intestine. Very High– Intense fluorescence extended from posterior intestine to anterior of vulva. Purple boxes indicate categories quantified in elevated expression analysis in Fig 2K and 2L. B: Quantification of percent of population in each category. Data from 3 pooled biological replicates. EV: Empty vector control. (ev RNAi till L4 then OP50 20°C, n = 86), (ev RNAi till L4 then PA14 20°C, n = 81), (tcer-1 RNAi till L4 then OP50 20°C, n = 83), (tcer-1 RNAi PA14 till L4 then 25°C, n = 78), (ev RNAi till L4 then OP50 25°C, n = 87), (ev RNAi till L4 then PA14 25°C, n = 81), (tcer-1 RNAi till L4 then OP50 25°C, n = 90), (tcer-1 RNAi till L4 then PA14 25°C, n = 72) C: Expression dynamics across lifespan. Representative images of lipl-2p:mCherry expression in (from left to right) young larvae, L4 larvae, newly-hatched young adult, Day 1 adult, and Day 5 adult worms. Pseudocolored in ImageJ with LUT Fire. (TIF) [file pgen.1011804.s005.tif]

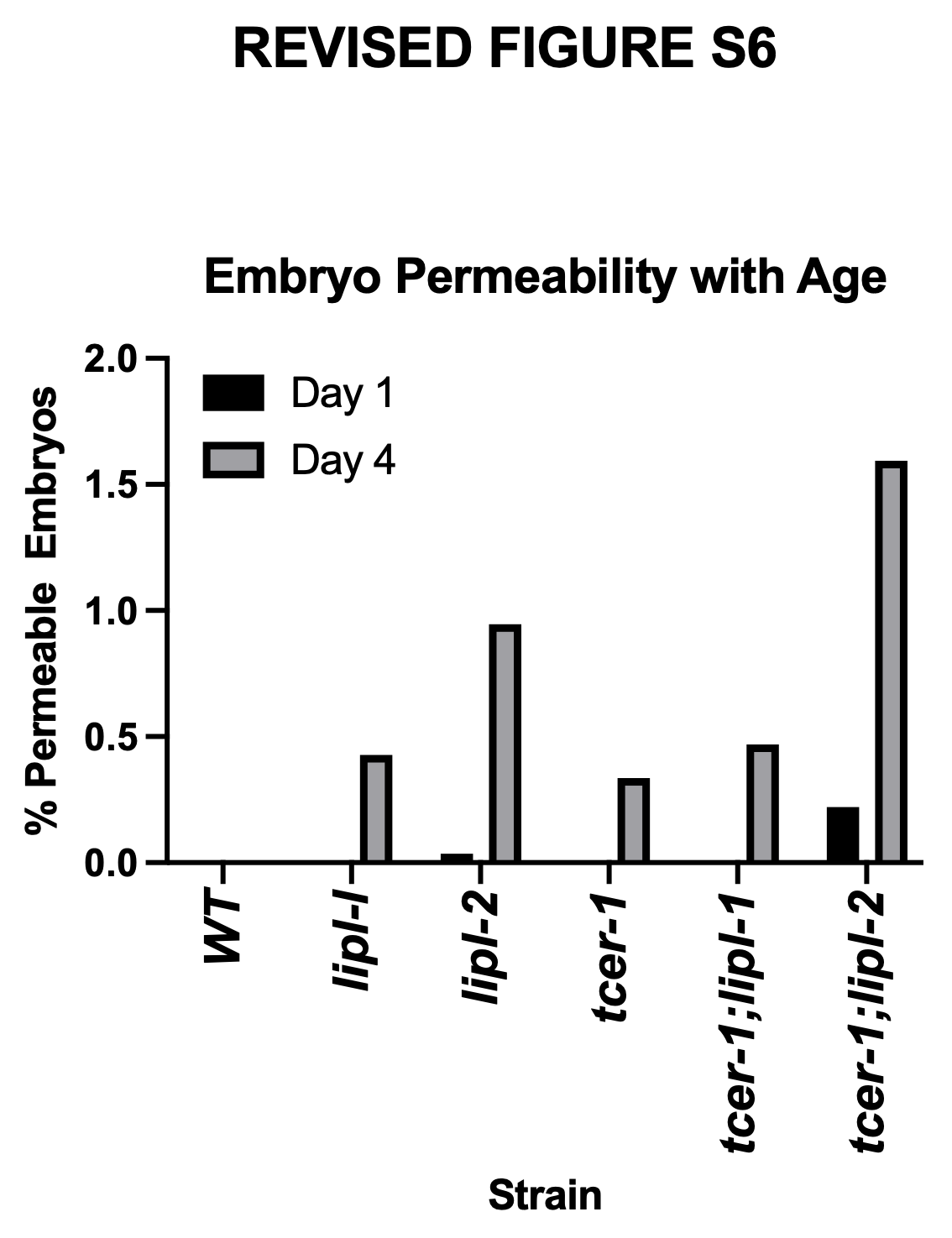

Supplement: S6 Fig — Longitudinal analysis of BODIPY-permeable eggs laid by Day 1 (D1) and Day 4 (D4) mothers by different strains. WT (D1 0, n = 712; D4 0, n = 276), lipl-l (D1 0, n = 700; D4 0.4267, n = 703), lipl-2 (D1 0.0356, n = 2806; D4 0.9451, n = 529), tcer-1 (D1 0, n = 896; D4 0.3361, n = 596), tcer-1;lipl-1 (D1 0, n = 1456; D4 0.4687, n = 640), tcer-1;lipl-2 (D1 0.2212, n = 1356; D4 1.594, n = 439). (TIFF) [file pgen.1011804.s006.tiff]

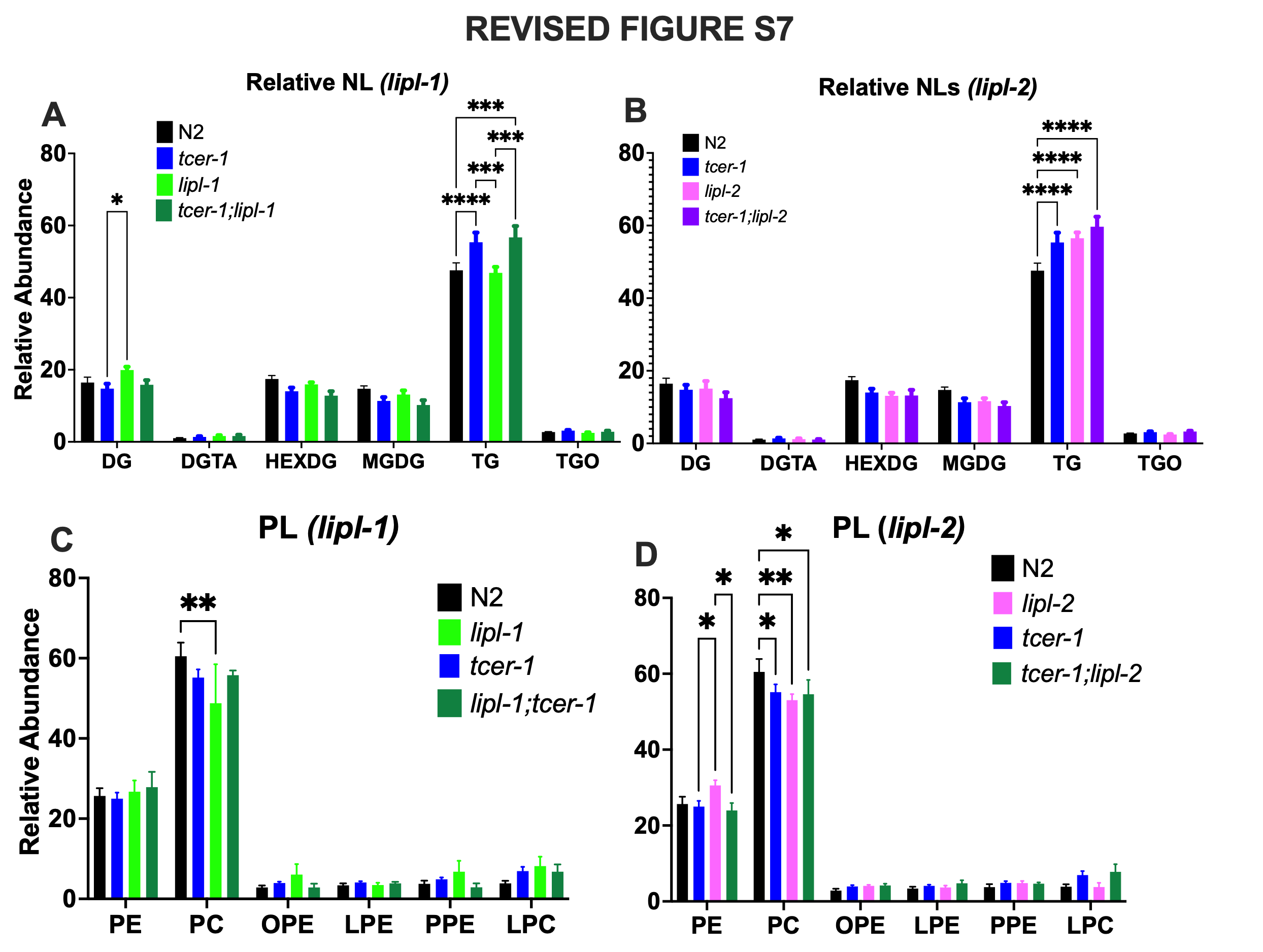

Supplement: S7 Fig — Lipids from gravid Day 1 adults were analyzed using HPLC-MS/MS. A, B: Impact of lipl-1 (A) or lipl-2 (B) inactivation on relative abundance of NLs with > 2% abundance in total NL population. C, D: Relative abundance of PL categories altered by lipl-1 (C) or lipl-2 (D) inactivation. Statistical significance was calculated using two-way ANOVA with Tukey’s correction, p ≤ 0.05(*), p < 0.01 (**), < 0.001 (***), < 0.0001 (****). (TIFF) [file pgen.1011804.s007.tiff]

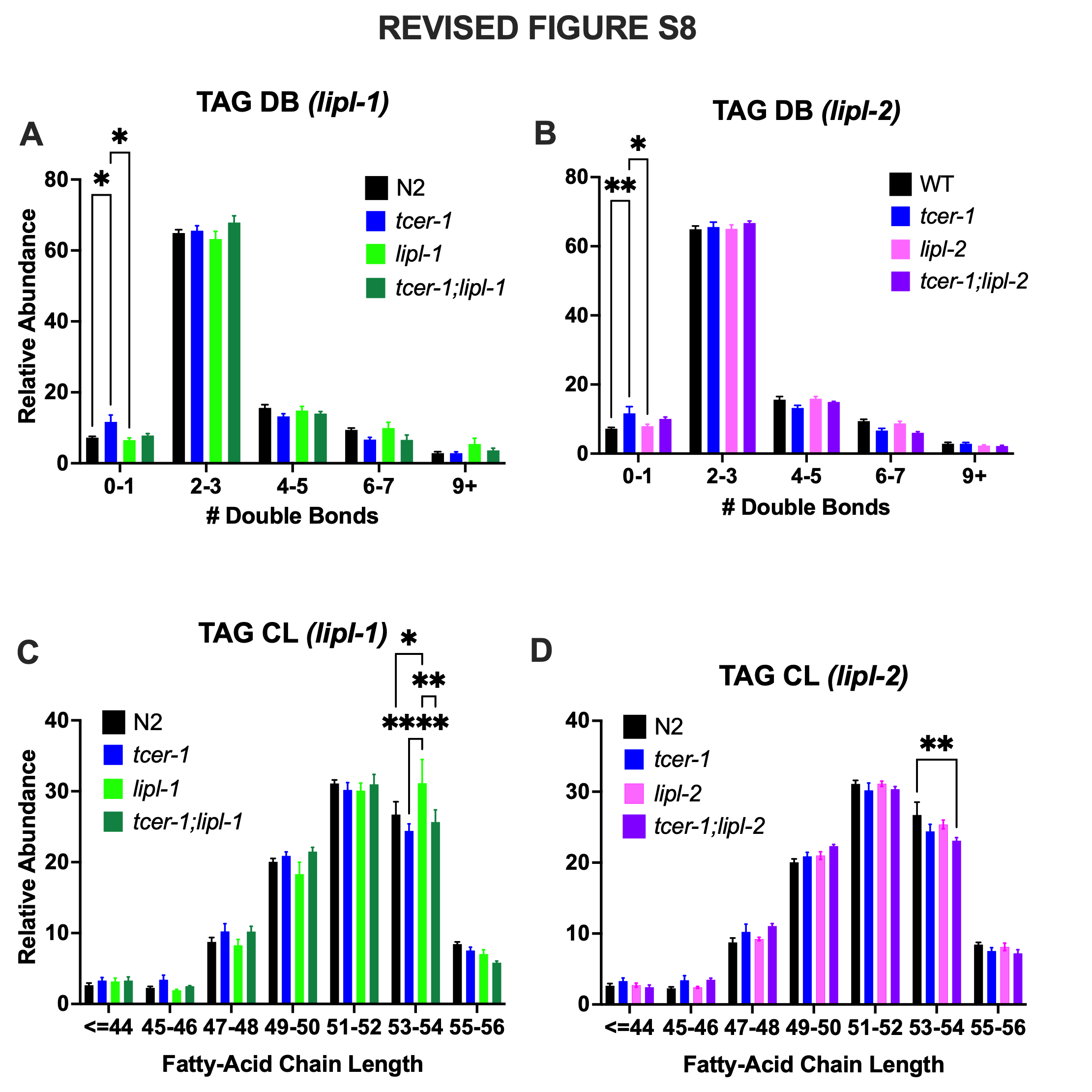

Supplement: S8 Fig — A, B: Relative abundance of double bonds (DB) in triacylglycerides (TAGs) altered by lipl-1 (A) or lipl-2 (B) inactivation. C, D: Relative abundance fatty-acid chain length (CL) of TAGs altered by lipl-1 (C) or lipl-2 (D) inactivation. Color key indicated on each panel. Statistical significance was calculated using two-way ANOVA with Tukey’s correction, p ≤ 0.05(*), p < 0.01 (**), < 0.001 (***), < 0.0001 (****). (TIFF) [file pgen.1011804.s008.tiff]

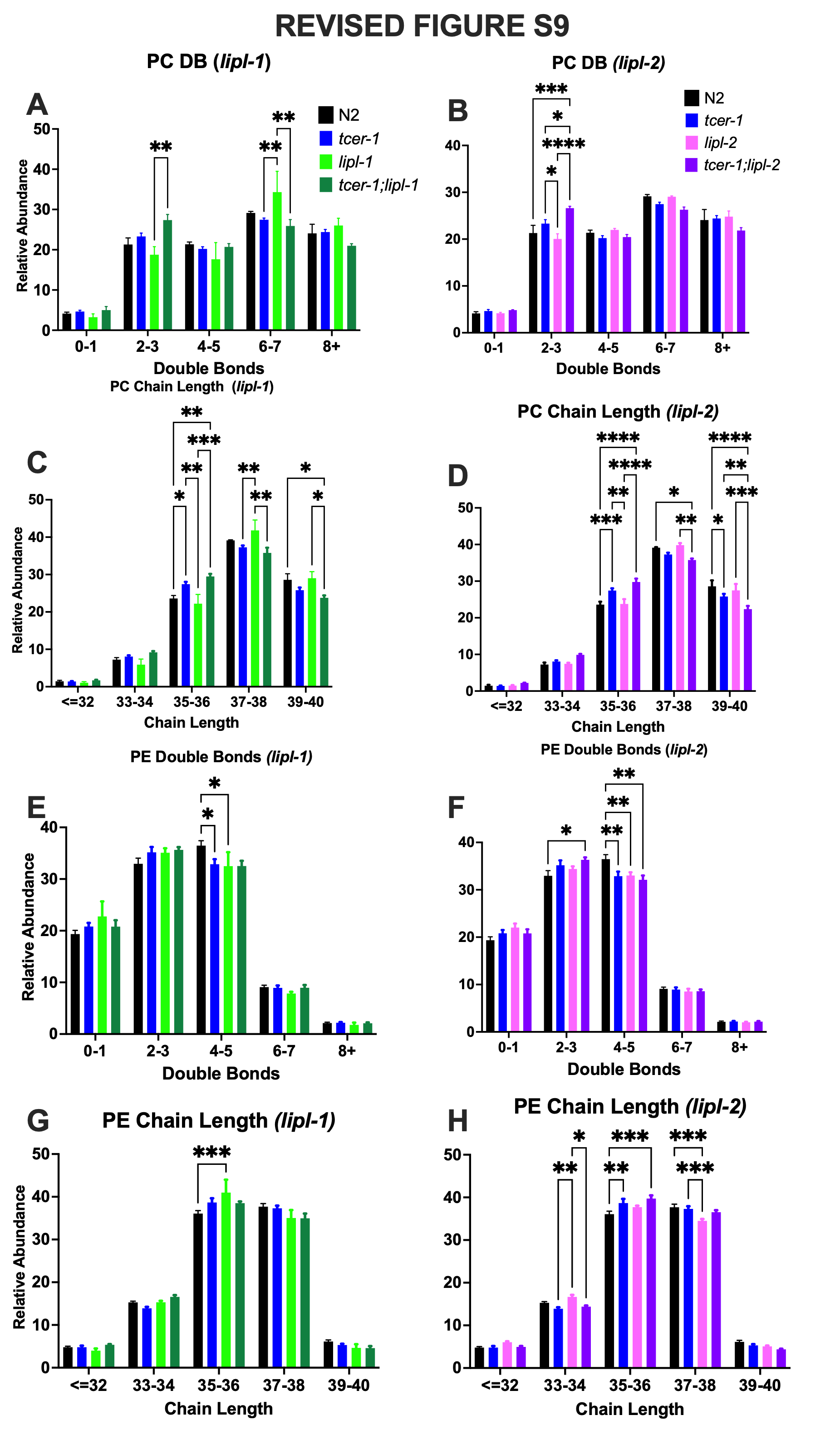

Supplement: S9 Fig — A, B: Relative abundance of PC double bonds (DB) altered by lipl-1 (A) or lipl-2 (B) inactivation. C, D: Relative abundance of PC chain length (CL) altered by lipl-1 (C) or lipl-2 (D) inactivation. E, F: Relative abundance of PE double bonds altered by lipl-1 (E) or lipl-2 (F) inactivation. G, H: Relative abundance of PE chain length altered by lipl-1 (G) or lipl-2 (H) inactivation. Color key of different strains shown at top. Statistical significance was calculated using two-way ANOVA with Tukey’s correction, p ≤ 0.05(*), p < 0.01 (**), < 0.001 (***), < 0.0001 (****). (TIFF) [file pgen.1011804.s009.tiff]

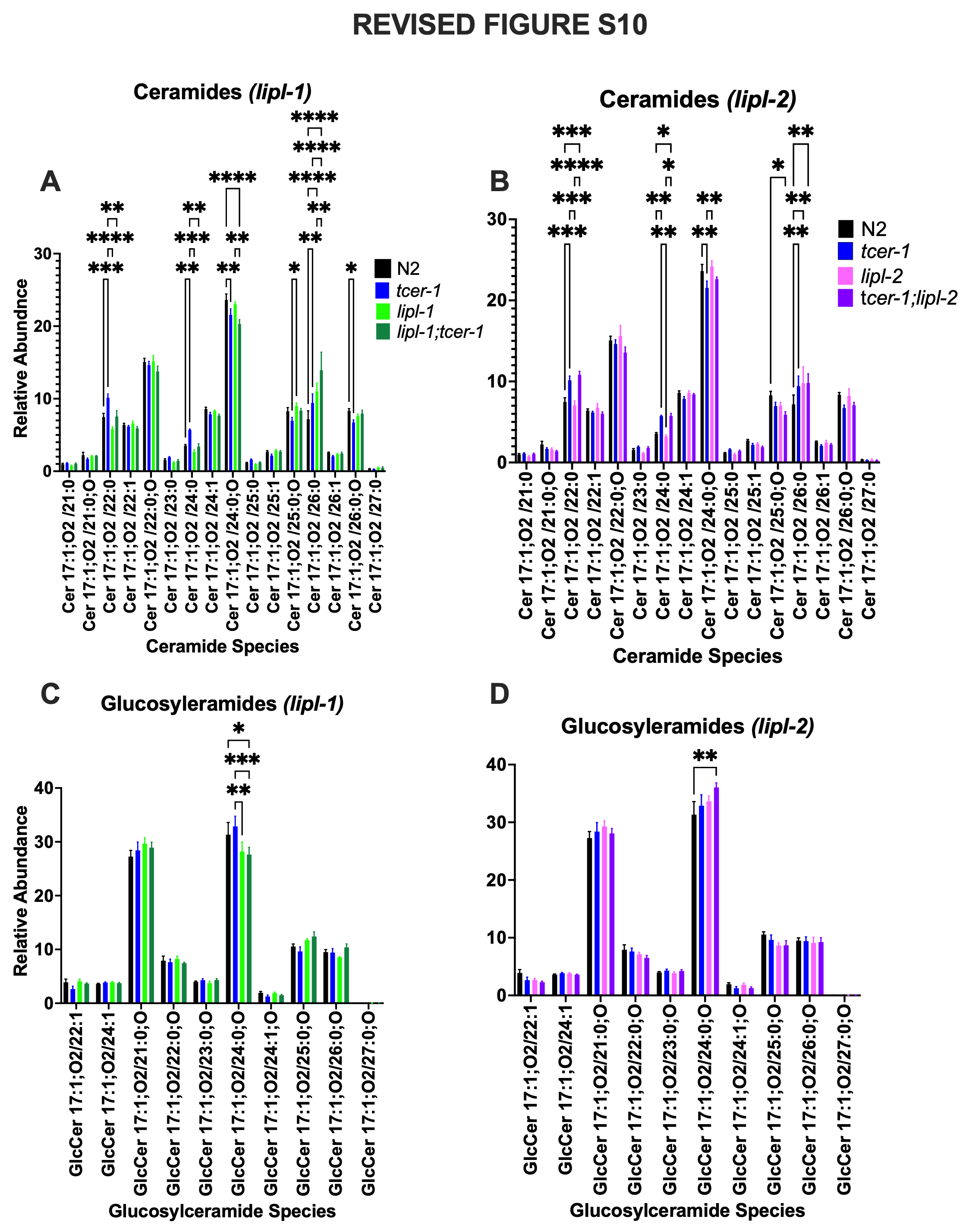

Supplement: S10 Fig — A, B: Relative abundance of Cers altered by lipl-1 (A) or lipl-2 (B) inactivation. C, D: Relative Abundance of GlcCers altered by lipl-1 (C) or lipl-2 (D) inactivation. Color key of different strains shown at top. Statistical significance was calculated using two-way ANOVA with Tukey’s correction, p ≤ 0.05 (*), p < 0.01 (**), < 0.001 (***), < 0.0001 (****). (TIFF) [file pgen.1011804.s010.tiff]
