# Supplementary material for: LIPL-1 and LIPL-2 are TCER-1-regulated lysosomal lipases with distinct roles in immunity and fertility
Source: PLoS Genet. 2025 Dec 12;21(12):e1011804. doi: 10.1371/journal.pgen.1011804 (PMC12716718; doi:10.1371/journal.pgen.1011804)
Supplement: S3 Table — (PDF) [file pgen.1011804.s013.pdf]

Table S3: Impact of *lipl-1* and *lipl-2* null mutants on survival upon *P. aeruginosa* PA14 infection.

***lipl-1* Strains**							
**Strain**	**Genotype**	**Trial 1**			**Bonferroni P-value**		
		**n = obs/ total**	**Mean**	**SE ^**	**P *(vs* N2)**	**P *(vs tcer-1*)**	**P *(vs lipl-1*)**
N2	WT	75/98	49.72	0.86			
CF2166	*tcer-1*	91/147	64.03	1.57	**0.00000002**		
AGP347	*lipl-1*	59/119	52.07	1.4	**0.0184**		
AGP354	*tcer-1;lipl-1*	75/135	56.32	1.57	**0**	0.1229	
**Trial 2**					**Bonferroni P-value**		
N2	WT	56/95	52.88	1.35			
CF2166	*tcer-1*	68/128	63.69	1.63	**0**		
AGP347	*lipl-1*	78/160	49.68	0.87	0.7954	**0.000000014**	
AGP354	*tcer-1;lipl-1*	101/126	48.17	0.8	1	**0**	
**Trial 3**					**Bonferroni P-value**		
N2	WT	41/50	56.64	2.05			
CF2166	*tcer-1*	100/120	71.59	2.04	**0.0001**		
AGP347	*lipl-1*	104/110	54.06	1.14	0.152		
AGP354	*tcer-1;lipl-1*	51/64	58.17	1.9	1	**0.0002**	
**Trial 4**					**Bonferroni P-value**		
N2	WT	76/93	52.58	1.41			
CF2166	*tcer-1*	68/100	66.56	1.55	**0.00000015**		
AGP347	*lipl-1*	77/94	56.29	1.19	1		
AGP354	*tcer-1;lipl-1*	73/95	59.76	1.34	0.1131	**0.0001**	
***lipl-2* Strains**							
**Strain**	**Background Genotype**	**Trial 5**			**Bonferroni P-value**		
		**n = obs/ total**	**Mean**	**SE ^**	**P *(vs* N2)**	**P *(vs tcer-1*)**	**P *(vs lipl-2*)**
N2	WT	102/119	59.27	1.21			
AGP364a	*lipl-2*	117/126	60.74	1.47	1		
AGP336a	*tcer-1*	98/123	67.44	1.51	**0**		
AGP358	*tcer-1;lipl-2*	84/127	75.27	1.85		**0.0045**	
AGP360a	*tcer-1; lipl-2 lipl-1*	124/138	55.56	1.34		**0**	**0**
**Trial 6**					**Bonferroni P-value**		
N2	WT	113/148	63.79	1.25			
AGP364a	*lipl-2*	107/123	72.55	2.15	0.1846		
AGP336a	*tcer-1*	123/134	91.69	2.25	**0**		
AGP358	*tcer-1;lipl-2*	100/129	95.53	3.07		1	
AGP360a	*tcer-1; lipl-2 lipl-1*	107/140	67.58	1.71		**0**	**0**
**Trial 7**					**Bonferroni P-value**		
N2	WT	86/113	51.13	1.32			vs. *lipl-1* /vs. *lipl-2*
AGP347	*lipl-1*	102/134	52.17	1.1	1		
AGP364a	*lipl-2*	61/123	70.64	1.5	**0**		
AGP357a	*lipl-2 lipl-1*	83/117	51.85	1.03	1		0.7811/**0**
AGP336a	*tcer-1*	87/112	70.61	2.09	**0**		
AGP354	*tcer-1;lipl-1*	85/120	56.04	1.23	**0.0004**	**0.0009**	
AGP358	*tcer-1;lipl-2*	66/124	78.23	2.66		1	
AGP360a	*tcer-1; lipl-2 lipl-1*	78/128	53.99	1.3		**0.000029**	**0.000024/0**
