# Supplementary material for: LIPL-1 and LIPL-2 are TCER-1-regulated lysosomal lipases with distinct roles in immunity and fertility
Source: PLoS Genet. 2025 Dec 12;21(12):e1011804. doi: 10.1371/journal.pgen.1011804 (PMC12716718; doi:10.1371/journal.pgen.1011804)
Supplement: S4 Table — (PDF) [file pgen.1011804.s014.pdf]

Table S4: Impact of *lipl-1* and *lipl-2* null mutants on lifespan on *E. coli* OP50.						
**Strain**	**Background Genotype**	**Trial 1**			**Bonferroni P-value**	
		**n = obs/ total**	**Mean**	**SE ^**	**P (*vs* N2)**	**P (*vs tcer-1*)**
N2	WT	77/121	19.03	0.77		
AGP347	*lipl-1*	79/116	17.4	0.65	0.537	
AGP364a	*lipl-2*	71/120	20.6	0.56	1	
AGP357	*lipl-1 lipl-2*	53/124	18.1	0.74	1	
AGP336a	*tcer-1*	65/118	15.87	0.74	0.0665	
AGP354	*tcer-1;lipl-1*	72/115	19.36	0.79		**0.0113**
AGP358	*tcer-1;lipl-2*	87/117	19.19	0.78		**0.0297**
AGP360a	*tcer-1;lipl-1 lipl-2*	82/117	15.75	0.5		1
**Trial 2**						
N2	WT	55/103	15.75	0.68		
AGP347	*lipl-1*	71/110	13.81	0.67	1	
AGP364a	*lipl-2*	52/103	15.8	0.96	1	
AGP357	*lipl-1 lipl-2*	64/100	11.95	0.66	**0.0004**	
AGP336a	*tcer-1*	39/103	12.08	0.76	**0.0031**	
AGP354	*tcer-1;lipl-1*	83/103	12.9	0.54		1
AGP358	*tcer-1;lipl-2*	79/97	15.31	0.69		**0.0369**
AGP360a	*tcer-1;lipl-1 lipl-2*	80/104	13.78	0.72		0.5519
**Trial 3**						
N2	WT	94/121	14.96	0.34		
AGP347	*lipl-1*	80/106	15.79	0.57	0.2334	
AGP364a	*lipl-2*	89/113	15.93	0.56	0.1141	
AGP357	*lipl-1lipl-2*	101/114	13.84	0.48	1	
AGP336a	*tcer-1*	92/120	14.23	0.48	1	
AGP354	*tcer-1;lipl-1*	77/120	15.36	0.51		0.9902
AGP358	*tcer-1;lipl-2*	81/103	16.72	0.58		**0.0133**
AGP360a	*tcer-1;lipl-1 lipl-2*	86/114	13.84	0.35		1
