# Supplementary material for: LIPL-1 and LIPL-2 are TCER-1-regulated lysosomal lipases with distinct roles in immunity and fertility
Source: PLoS Genet. 2025 Dec 12;21(12):e1011804. doi: 10.1371/journal.pgen.1011804 (PMC12716718; doi:10.1371/journal.pgen.1011804)
Supplement: S5 Table — (PDF) [file pgen.1011804.s015.pdf]

Table S5: Lipid species altered in *tcer-1* mutants and impacts of *lipl-1* and *lipl-2* mutations on them.

Lipid Categories			WT vs *tcer-1*		*tcer-1;lipl-1* vs *tcer-1*		*tcer-1;lipl-2* vs *tcer-1*	
	**Major Class**	**Species**	***tcer-1* /WT Ratio**	**Statistical significance (q value)**	***tcer-1;lipl-1* /*tcer-1* Ratio**	**Statistical significance (q value)**	***tcer-1;lipl-2* /*tcer-1* Ratio**	**Statistical significance (q value)**
**Neutral Lipids**	**TG**		1.15	****	no significant changes			
	**MGDG**	MGDG 32:2	1.565737052	0.007177	no significant changes		no significant changes	
		MGDG 38:5	0.616957606	0.007177				
		MGDG 38:4	0.483273056	0.026845				
		MGDG 36:11	0.910572503	0.027074				
	**HEXDG**	HEXDG 37:2	0.464132231	0.003409	no significant changes		no significant changes	
		HEXDG 37:7	1.417880795	0.003409				
		HEXDG 37:9	0.750509857	0.005333				
		HEXDG 37:10	0.451458753	0.030091				
		HEXDG 37:4	0.714666021	0.034491				
**Phospho-lipids**	**PC**	PC 37:6	0.711318272	0.000803	no significant changes		no significant changes	
		PC 39:4	0.473088837	0.001716				
		PC 38:7	1.42758289	0.001716				
		PC 39:6	0.620129221	0.00195				
		PC 38:2	0.487076948	0.003067				
		PC 33:2	1.582312075	0.005082				
		PC 36:4	1.553013469	0.006053				
		PC 36:3	1.771081002	0.006053				
		PC 37:4	0.649282564	0.006981				
		PC 39:5	0.631199353	0.006981				
		PC 32:2	1.43658948	0.008009			1.782691575	0.00098
		PC 38:4	0.832043425	0.02792			no significant changes	
	**LPC**	LPC 18:2	1.653066306	0.000388	no significant changes		1.292298424	0.018886
		LPC 17:1	0.678693403	0.001427			no significant changes	
		LPC 19:1	0.538062535	0.001427				
	**PE**	PE 39:6	0.533642249	0.001592	no significant changes		no significant changes	
		PE 40:4	0.546087105	0.001592				
		PE 36:2	1.309779671	0.015054				
		PE 37:6	0.734816912	0.021557				
		PE 36:1	1.355844676	0.02955				
		PE 36:4	0.838629509	0.034792				
		PE 35:4	0.785869456	0.038881				
		PE 40:2	1.911385285	0.038881				
		PE 34:3	0.75335599	0.038881				
		PE 37:4	0.814092018	0.043916				
**Sphingo-lipids**	**Cer**	**Cer 17:1;O2 /22:0**	**1.355226491**	**0.0002**	**0.743967625**	**0.0032**	no significant changes	
		**Cer 17:1;O2 /24:0**	**1.613143178**	**0.0036**	**0.589059338**	**0.0097**		
		Cer 17:1;O2 /24:0; O	0.912544262	0.0065	no significant change			
		**Cer 17:1;O2 /26:0**	**1.310880231**	**0.0026**	**1.478907888**	**<0.0001**		
		Cer 17:1;O2 /26:0;O	0.804705029	0.0496	no significant change			
	**GlcCer**	no significant species changes						
		* p-value						
