# Supplementary material for: LIPL-1 and LIPL-2 are TCER-1-regulated lysosomal lipases with distinct roles in immunity and fertility
Source: PLoS Genet. 2025 Dec 12;21(12):e1011804. doi: 10.1371/journal.pgen.1011804 (PMC12716718; doi:10.1371/journal.pgen.1011804)
Supplement: S6 Table — (PDF) [file pgen.1011804.s016.pdf]

Table S6: Lipid species altered in *lipl-1* and *lipl-2* mutants

Lipid Categories			WT vs *lipl-1*		WT vs *lipl-2*	
	**Major Class**	**Species**	***lipl-1* /WT Ratio**	**Statistical significance (q value)**	***lipl-2* /WT Ratio**	**Statistical significance (q value)**
**Neutral Lipids**	**TG**	no significant changes				
	**MGDG**	no significant changes				
	**HEXDG**	no significant changes				
**Phospho-Lipids**	**PC**	no significant changes				
	**LPC**	no significant changes				
	**PE**	no significant changes				
**Sphingo- Lipids**	**Cer**	Cer 17:1;O2 /26:0	1.534223979	<0.0001	**1.360074367**	**0.0032**
		Cer 17:1;O2 /22:0	no significant changes		no significant changes	
	**GlcCer**	no significant changes				
