# Supplementary material for: LIPL-1 and LIPL-2 are TCER-1-regulated lysosomal lipases with distinct roles in immunity and fertility
Source: PLoS Genet. 2025 Dec 12;21(12):e1011804. doi: 10.1371/journal.pgen.1011804 (PMC12716718; doi:10.1371/journal.pgen.1011804)
Supplement: S7 Table — (PDF) [file pgen.1011804.s017.pdf]

Table S7A: Impact of Cer 24 and LPC18:2 Supplementation on PA14 Response							
**Trial 1**							
**Strain**	**Genotype**	**Treatment**	**Survival Data**			**Bonferroni P-value**	
			**n = obs/ total**	**Mean**	**SE ^**	**P *(vs* N2 ctrl)**	**P *(vs tcer-1; lipl-1*)**
N2	WT	Ethanol	74/90	63.09	1.53		
N2	WT	Cer 24	58/90	70.57	1.78	0.0032	
AGP354	*tcer-1;lipl-1*	Ethanol	82/90	58.1	1.33		
AGP354	*tcer-1;lipl-1*	Cer 24	71/90	73.74	2.01		<0.0001
N2	WT	LPC 18:2	65/77	61.60	1.79	0.5759	
**Trial 2**							
			**Survival Data**			**Bonferroni P-value**	
N2	WT	Ethanol	73/90	68.41	2.02		
N2	WT	Cer 24	76/90	75	2.04	0.092	
AGP354	*tcer-1;lipl-1*	Ethanol	75/90	60.55	1.57		
AGP354	*tcer-1;lipl-1*	Cer 24	78/90	67.76	1.96		0.0197
N2	WT	LPC 18:2	75/90	64.12	1.71	0.2425	
**Table S7B: Impact of FuDR Treatment on PA14 Response**							
**Trial 1**							
			**Survival Data**			**Bonferroni P-value**	
N2	WT	FuDR	124/128	66.07	1.02		
CF2166	*tcer-1*	FuDR	106/122	80.01	1.67	<0.0001	
N2	WT	No FuDR	64/153	62.22	1.61		
Cf2166	*tcer-1*	No FuDR	115/152	79.84	2.06	1.3E -08	
**Trial 2**							
			**Survival Data**			**Bonferroni P-value**	
N2	WT	FuDR	106/123	67.52	1.2		
CF2166	*tcer-1*	FuDR	97/116	102.94	3.32	<0.0001	
N2	WT	No FuDR	113/122	57.02	1.07		
Cf2166	*tcer-1*	No FuDR	91/137	89.15	2.48	<0.0001	
