# Supplementary material for: LIPL-1 and LIPL-2 are TCER-1-regulated lysosomal lipases with distinct roles in immunity and fertility
Source: PLoS Genet. 2025 Dec 12;21(12):e1011804. doi: 10.1371/journal.pgen.1011804 (PMC12716718; doi:10.1371/journal.pgen.1011804)
Supplement: S8 Table — (PDF) [file pgen.1011804.s018.pdf]

**Table S8: Survival of strains expressing human LAL (hLAL/LIPA) in different genetic backgrounds.**							
**Strain**	**Genotype**	**Trial 1***			Bonferroni P-value		
		**n = obs/ total**	**Mean**	**SE ^**	P (vs WT)	*P (vs tcer-1)*	*P (vs. tcer-1;lipl-1)*
WT	WT	54/90	35.06	1.91		0.0000034	1
CF2166	*tcer-1*	63/90	54.8	3.25	0.0000034		0.000021
AGP354	*tcer-1;lipl-1*	52/90	36.65	1.79	1	0.000021	
AGP368a	*tcer-1;lipl-1;hLAL (A)*	73/90	55.77	2.53	<0.0001	1	<0.0001
AGP368b	*tcer-1;lipl-1;hLAL (B)*	70/90	40.14	1.68	0.0738	0.0002	0.5904
**Trial 2**							
N2	WT	51/90	40.29	2.7		<0.0001	0.0000027
CF2166	*tcer-1*	54/90	68.56	2.99	<0.0001		0.0126
AGP354	*tcer-1;lipl-1*	42/90	55.31	4.57	0.0000027	0.0126	
AGP368a	*tcer-1;lipl-1;hLAL (A)*	43/90	63.53	2.59	<0.0001	0.4523	0.0866
AGP368b	*tcer-1;lipl-1;hLAL (B)*	39/90	63.08	3.66	<0.0001	0.6723	0.1765
**Trial 3**							
N2	WT	40/90	64.46	4.8		<0.0001	0.0000027
CF2166	*tcer-1*	52/90	91.96	4.08	<0.0001		0.0126
AGP354	*tcer-1;lipl-1*	32/90	71.35	4.61	0.0000027	0.005	
AGP368b	*tcer-1;lipl-1;hLAL (B)*	47/90	79.14	3.67	<0.0001	0.661	0.47
COP2593	hLAL	35/90	81.76	2.33	<0.0001		
**Trial 4****							
N2	WT	35/110	57.24	2.01			
COP2593	hLAL	46/96	83.18	2.22	<0.0001		
COP2589	hLAL	63/103	82.84	2.22	<0.0001		
**Trial 5**							
N2	WT	41/90	43.87	2.08			
COP2593	hLAL	31/90	53.44	1.75	<0.0001		
COP2589	hLAL	28/90	51.79	2.31	0.0000027		
** Shown in Figure 7A and ** 7B*							
