# Supplementary material for: LIPL-1 and LIPL-2 are TCER-1-regulated lysosomal lipases with distinct roles in immunity and fertility
Source: PLoS Genet. 2025 Dec 12;21(12):e1011804. doi: 10.1371/journal.pgen.1011804 (PMC12716718; doi:10.1371/journal.pgen.1011804)
Supplement: S9 Table — (PDF) [file pgen.1011804.s019.pdf]

Table S9: Strains used in this study.

Strain name	Genotype	Transgene/Description	Description/Comment	Source
N2	WT			
AGP334	N2	*[Plipl-1(441bp)::mCherry + Pmyo-3::GFP] 50ng/ul, 15ng/ul*	mCherry driven under 441bp *lipl-1* promoter	This study
AGP340	N2	*[Plipl-1(1kb)::mCherry + Pmyo-3::GFP] 50ng/ul, 15ng/ul*	mCherry driven under 1016bp *lipl-1* promoter	This study
AGP335	N2	*[Plipl-2(1.5kb)::mCherry + Pofm-1::GFP] 50ng/ul, 15ng/ul*	mCherry driven under 1.5kb *lipl-2* promoter	This study
AGP342	N2	*[Plipl-2(1kb)::mCherry + Pmyo-3::GFP] 50ng/ul, 50ng/ul*	mCherry driven under 1kb *lipl-2* promoter	This study
AGP341	N2	*[Plipl-2::LIPL-2::mRFP + Pmyo-3::GFP] 25ng/ul, 15ng/ul*	LIPL-2::RFP overexpression	This study
AGP339	N2	*[Plipl-1(1kb)::LIPL-1::mRFP + Pmyo-3::GFP] 25ng/ul, 15ng/ul*	LIPL-1::RFP overexpression	This study
COP2589	EG6699/COP93	*knuSi924 [pNU3447 (eft-3p::hLIPA::linker::wrmScarlet::3xFLAG::tbb-2u in cxTi10882, unc-119(+))] IV ; unc-119(ed3) III*	Human LAL expressed broadly in soma	This study
COP2593	EG6699/COP93	*knuSi927 [pNU3447 (eft-3p::hLIPA::linker::wrmScarlet::3xFLAG::tbb-2u in cxTi10882, unc-119(+))] IV ; unc-119(ed3) III*	Human LAL expressed broadly in soma	This study
VS56	N2	*hj340 [cpg-2 signal peptide::mCherry_TEV_3xFLAG::cpg-2] ltls38 [pie-1p::GFP::PH(PLC1delta1) + unc-119(+)]*	mCherry::CPG-2 and GPF::PH(PLC1delta1) ex	gift from Dr. Ho Yi Mak
CF2166	*tcer-1*	*tcer-1(tm1452) II*		
AGP336a	*tcer-1*	*tcer-1(tm1452)/mnC1 [dpy-10(e128) unc-52(e444)] II.*		This study
AGP347	*lipl-1*	*full crispr deletion*		This study
AGP364a	*lipl-2*	*full crispr deletion*		This study
AGP357a	*lipl-2 lipl-1*	*full crispr deletion both lipases*		This study
AGP354	*tcer-1;lipl-1*	*tcer-1(tm1452) crossed to lipl-1 Crisper null*		This study
AGP358	*tcer-1;lipl-2*	*tcer-1(tm1452) crossed to lipl-2Crisper null*		This study
AGP360a	*tcer-1; lipl-2 lipl-1*	*tcer-1(tm1452) crossed to lipl-1 lipl-2 Crisper double null*		This study
AGP368b	*tcer1;lipl-1;hLAL*	*COP293 crossed into tcer-1;lipl-1 mutant strain*		This study
