# Supplementary material for: LIPL-1 and LIPL-2 are TCER-1-regulated lysosomal lipases with distinct roles in immunity and fertility
Source: PLoS Genet. 2025 Dec 12;21(12):e1011804. doi: 10.1371/journal.pgen.1011804 (PMC12716718; doi:10.1371/journal.pgen.1011804)
Supplement: S10 Table — (PDF) [file pgen.1011804.s020.pdf]

Table S10: Primers used in this study.

Gene	direction	Sequence
**Diagnostic Genotyping**		
lipl-2	F	TGCACAAAGAGTTTGGCGTT
	R1	TCCCCATCGTTCAATGATTTGACT
	R2	AGTCCGGTTGATGCGACAAA
lipl-1	F	CCGTGACTGCGTTTTTCGTT
	R1	GACCTGGGGCACTCACTTTT
	R2	TGTGTTAACCGTGTGACTCCT
tcer-1	F	gccaattctggttgagtgac
	R	TCGTCTTGACTGATGGACAC
**QPCR**		
lipl-1	F	GGACTTAAAGTTGAAGCTGGAG
	R	AACACGAGTTGCGTTAAGC
lipl-2	F	GTTACATGGCCAAATGGGA
	R	AAACGAAAGCTGCACTCTG
rpl-32	F	GGATTTGGACATGCTCCTC
	R	GATTCCCTTGCGGCTCTT
Y45F10D.4	F	TTCACTGTTCAATGCTCGC
	R	CTTAGGCCTTCTTAGTCTGCT
**Cloning**		
lipl-2 1.5kb	F	gattacgccaagcttgcatgactatgagggccgtttcaattc
	R	cctcgcccttgctcaccatgtttgttctagtacgatcagttatcattac
lipl-1 441 kb	F	gattacgccaagcttgcatggtggaaatatttcagaaactttc
	R	cctcgcccttgctcaccatgttcaaccagagtctacctg
lipl-1 1kb	F	gattacgccaagcttgcatgaaaattgaaactttcgccac
	R	cctcgcccttgctcaccatgttcaaccagagtctacctg
lipl-2 1kb	F	gattacgccaagcttgcatgAATCATGAAAGTATAATAATTGAGG
	R	cctcgcccttgctcaccatgTTTGTTCTAGTACGATCAG
lipl-1::RFP	F	actatagggcgaattggggtaccagcttcaataaaaattgaaactttc
	R	tcggaggaggccatcccgggggaaccgttcaaaac
**CRISPR-Cas9 Oligos**		
dpy-10 co-CRISPR	repair template 1	CACTTGAACTTCAATACGGCAAGATGAGAATGACTGGAAACCGTACCGCAT
	repair template 2	GCGGTGCCTATGGTAGCGGAGCTTCACATGGCTTCAGACCAACAGCCTAT
	gRNA	GCUACCAUAGGCACCACGAGGUUUUAGAGCUAUGCU
lipl-1 target gRNA	F	AUUGUGCACAAAUUAAGGACGUUUUAGAGCUAUGCU
	R	CAUUAUUAUGUUCUUUUAACGUUUUAGAGCUAUGCU
	repair template	ATGGGCGGGCACAGTAATAATTGTGCACAAATTAAAAAGAACATAATAATGTTCTCAAAATGTTATTTTA
lipl-2 target gRNA	F	GUUUAAGACUACAAUGUACUGUUUUAGAGCUAUGCU
	R	ACAGCUAAGGAUUAGAUCAUGUUUUAGAGCUAUGCU
	repair template	CTTCTAAATTCAGAGAAAATCTAAATTCTCCCCAAGTCATTGGAAAAGGAAGCAATGACAAAAATAGATCAC
