# Supplementary material for: LIPL-1 and LIPL-2 are TCER-1-regulated lysosomal lipases with distinct roles in immunity and fertility
Source: PLoS Genet. 2025 Dec 12;21(12):e1011804. doi: 10.1371/journal.pgen.1011804 (PMC12716718; doi:10.1371/journal.pgen.1011804)
Supplement: Attachment — Submitted filename: BahrEtal_Resp_Reviewers.pdf [file pgen.1011804.s021.pdf]

RESPONSE TO REVIEWERS

We are sincerely grateful to the reviewers for their insightful and constructive feedback, which has substantially strengthened the clarity and rigor of our manuscript. Below we provide detailed, point-by-point responses addressing each of their comments (in blue).

Reviewer #1

The study by Bahr et al. identifies LIPL-1 and LIPL-2 as lysosomal lipases functioning downstream of TCER-1, a transcriptional and splicing regulator that promotes fertility while suppressing immunity in C. elegans. Through transcriptomic, lipidomic, and genetic analyses, the authors demonstrate that these lipases have distinct, context-dependent roles in immunity, reproduction, lipid metabolism, and lifespan: LIPL-1 enhances immune resistance, whereas LIPL-2 primarily supports fertility. Importantly, the human ortholog LAL rescues lipl-1–associated immune defects, suggesting an evolutionarily conserved role in host defense. **This work advances our understanding of conserved lipases in regulating immunity and development.**

I have several suggestions/questions to further improve the manuscript:

We sincerely thank the reviewer for their recognition and appreciation of our work's significance, and for offering thoughtful suggestions that enriched and extended this study.

1.Are the translational reporters used in this study functional? Could the different promoter lengths used in various constructs influence the observed expression patterns?

We created the transcriptional and translational reporter strains with two promoter fragments for each gene. For *lipl-1*, we used a previously described 441bp promoter (*O'Rourke and Ruvkun, Nat. Cell. Biol. 2013; PMID: 23604316*) as well as a transgene driven by 1kb of upstream promoter region. We did not observe a manifest difference in expression patterns between the two. For the *lipl-2* transcriptional reporters, we used 1kb (based on a previous construct described below) and 1.5kb promoters. These strains did not exhibit major expression differences either. While we cannot exclude the possibility that further promoter analyses may provide greater clarity regarding the *lipl's* expression domains, we chose not to expand the promoter regions further to prevent interference from neighboring genes that would have been included in the constructs of longer sizes.

One LIPL-2 translational reporter we used (*lipl-2p::lipl-2::rfp*; 1468bp of upstream promoter sequence linked to *lipl-2* genomic DNA) we re-created is a functional construct previously used to assess the role of the enzyme in starvation response, created by Abhinav Diwan's lab (*Murphy et al., PLoS Biol. 2019; PMID: 31086360*). The second was driven by 1kb promoter. We created LIPL-1 translational reporters too using both the 441bp and 1kb promoter fragments. However, we were unable to directly test the functionality of these strains in our survival or fertility assays because they could not be stabilized/maintained easily across generations, showed significant developmental variability and reduced fertility. It is likely this can be attributed to the array-based construction of the transgenic strains. This information has now been added to the manuscript for clarity (Pg. 25, Lines 483-485).

2. The study reports lipl transgene expression in multiple tissues. In which tissues are lipl-1 and lipl-2 expression required and sufficient to produce the observed phenotypes?

We thank the reviewer for this insightful question. We are actively interested in identifying the precise tissue- and sub-cellular sites of action for these genes. However, the variability in their expression both across tissues (e.g., neurons vs. intestine) and within tissues (anterior, mid-, and posterior intestinal regions) currently limits the feasibility of traditional tissue-specific gain- or loss-of-function approaches. Given the constraints of available site-specific overexpression and RNAi drivers, such experiments would likely yield ambiguous results. Defining these site-specific roles remains a key focus of our ongoing and future studies.

3. Can supplementation with any lipid species identified in the lipidomics analysis rescue the infection or reproductive phenotypes in the mutant backgrounds?

We thank the reviewer for this suggestion. We were able to test two lipid species in supplementation experiments. (i) the Ceramide, C17:1; O2/24:0 (Cer 24) that is elevated in *tcer-1* mutants in a *lipl-1*-dependent manner, and (ii) the lysophosphatidyl choline, LPC 18:2, which is elevated in *tcer-1* mutants and further increased in *tcer-1;lipl-2* double mutants. As shown below, Cer 24 supplementation completely rescued the survival of *tcer-1;lipl-1* mutants to *tcer-1* levels. Additionally, WT animals supplemented with Cer 24 survived significantly longer upon PA14 infection. Since LPC 18:2 levels correlated with immunoresistance, we also asked if its supplementation could extend survival of WT animals on PA14, but it did not. These data are shown in Figure 5J and K and Table S7)

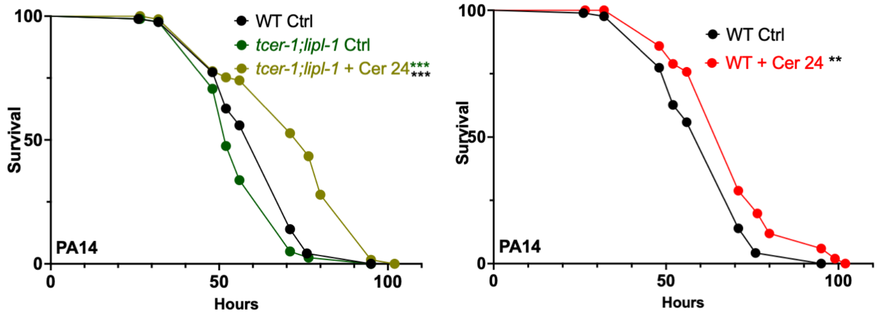

Table S: Impact of Cer 24 and LPC18:2 supplementation on *P. aeruginosa* PA14 response.

Trial 1

Strain	Background Genotype	Supplementation	Survival Data: n = obs/ total	Survival Data: Mean	Survival Data: SE ^	Bonferroni P-value: P (vs N2 ctrl)	Bonferroni P-value: P (vs lipl-1;tcer-1 Ctrl)
N2	WT	Ethanol	74/90	63.09	1.53		
N2	WT	Cer 24	58/90	70.57	1.78	0.0032	
AGP354	*tcer-1;lipl-1*	Ethanol	82/90	58.1	1.33		
AGP354	*tcer-1;lipl-1*	Cer 24	71/90	73.74	2.01		<0.0001
N2	WT	LPC 18:2	65/77	61.60	1.79	0.5759	

4. Can the human homolog hLAL functionally replace lipl-2 as well? Does it also support reproductive function?

We conducted several experiments to determine whether the human hLAL/LIPA could rescue the fertility defects associated with *lipl-1* or *lipl-2* inactivation. As shown below, while hLAL/LIPA expression appeared to improve some fertility measures, its effects were inconsistent across experimental replicates and genetic backgrounds. For example, *hLAL* expression produced a modest increase in progeny number *tcer-1;lipl-2* mutants, but this trend did not reach statistical significance (**D below**). A similar pattern was seen for the embryonic permeability phenotype- there was a trend towards reduction in the number of porous embryos in *tcer-1;lipl-2* but it was not statistically relevant (**E**). Taken together, we chose the parsimonious interpretation that hLAL

has inconsistent impact on fertility measures and does not show a significant rescue of reproductive phenotypes associated with *lipl-1* or *lipl-2* inactivation. These data are now shown in Fig. 6C-E.

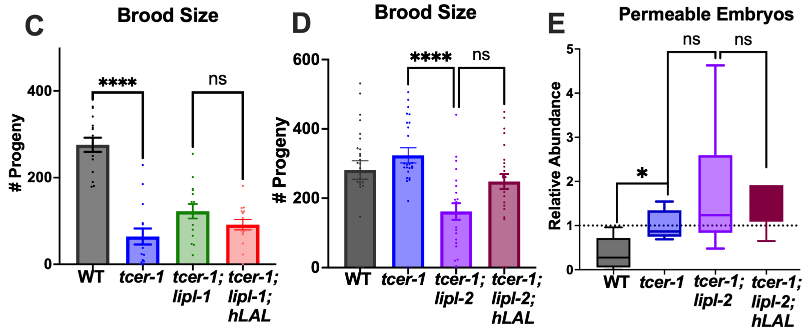

5. Adding a model figure summarizing the effects of each gene on immunity, reproduction, and lifespan would help synthesize the findings for readers.

We thank the reviewer for this suggestion and have included a summary figure (Fig. 7).

FIGURE 7

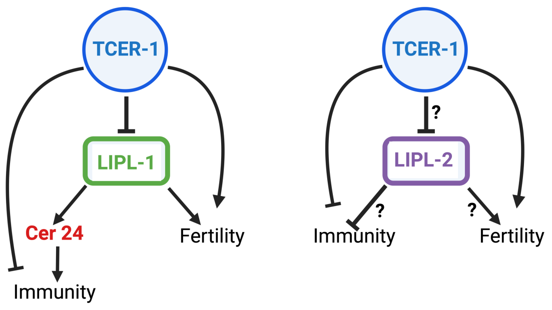

Figure 7. Schematic depicting the regulation of lysosomal lipases, *lipl-1* and *lipl-2*, by TCER-1 and their distinct roles in immunity and fertility identified in this study. TCER-1 represses the expression of *lipl-1* upon infection, which in turn modulates levels of the specific fatty-acid species, such as the Ceramide 24 (Cer 24) that promote immune resilience. *lipl-1* also acts with TCER-1 to promote fertility (left panel). In contrast, the relationship between TCER-1 and *lipl-2* appears more complex. Although TCER-1 represses *lipl-2* induction upon infection, both genes repress immunity and promote fertility, suggesting that TCER-1 may act through additional pathways to coordinate these processes (right panel). Arrows denote activation, and blunt-ended lines denote repression. Figure created using Biorender.

6. There are inconsistencies in labeling double and triple mutants; the order of gene names should follow their chromosomal positions. tcer-1 is on chromosome II, while lipl-2 and lipl-1 are on chromosome V, with lipl-2 located earlier on the genetic map and therefore listed first. Promoter nomenclature should follow standard conventions, using gene name plus a lowercase p rather than a capital P.

We apologize for these oversights and thank the reviewer for bringing them to our attention. The nomenclature has been corrected across the manuscript and in figures.

7. Replace "normal" animals with "uninfected" animals for more precise language.

Corrected

8. Remove references to "data not shown," as PLOS does not permit this.

Corrected. Thank you for pointing out the journal policy.

Overall, this is an interesting and well-executed study. Addressing the points above would improve clarity, reproducibility, and adherence to journal standards.

We would like to thank the reviewer for the encouraging comments and valuable suggestions.

Reviewer #2

In this manuscript, Bahr et al. find that the expression of two lysosomal lipases, lipl-1 and lipl-2, changes in tcer-1 mutant animals, and show that these two genes have distinct activities and contribute to different tcer-1 mutant phenotypes. The authors identified lipl-1 and lipl-2 using RNAseq of tcer-1 mutants that were challenged with pathogenic Pseudomonas aeroginosa (PA). The abundance of the two lipl transcripts increase in wild-type animals challenged with PA. The induction of lipl genes is exaggerated in tcer-1 mutants, even though basal expression on non-pathogenic OP50 is reduced. The authors show that lipl-1 and lipl-2 mRNA are expressed in the intestine but that these proteins localize to coelomocytes, consistent with previous reports and studies of other lipl genes in C. elegans. Phenotypic analysis shows that lipl-1 is required for the increased survival of tcer-1 mutants when challenged with PA but does not change the survival of wild-type animals on PA. In contrast, loss of lipl-2 itself confers resistance to PA in wild-type animals similar to tcer-1 and there is no additive effect in the tcer-1; lipl-2 double mutant. The effects of lipl genes is specific to PA, as the lifespan of single and double mutants on OP50 was indistinguishable from wild-type. The authors also investigated the role of these lipl genes in fertility, as tcer-1 mutants are known to have reduced fertility. The authors show that loss of either lipl-1 or lipl-2 further reduces the number of progeny produced by tcer-1 mutants, but have no effect in the wild-type background. Closer examination showed that loss of either lipl gene increased embryonic lethality and sterility in tcer-1 mutants, and that loss of lipl-2 (but not lipl-1) increases the fraction of embryos with an intact permeability barrier. The authors next examine how the loss of the lipl genes changes the lipid profile of tcer-1 animals. The lipl genes are orthologues of the human lysosomal acid lipase (hLAL), and the authors show that hLAL can rescue the immunity defect of lipl-1 animals, suggesting some functions are conserved.

Previous work from this group has demonstrated that TCER-1, an orthologue of the human transcription elongation and splicing factor TRCERG1, coordinates immunity, longevity, and fertility. This work shows that the effects of TCER-1 on immunity and fertility are mediated, in part, by regulation of LIPL-1 and LIPL-2. Moreover, the authors demonstrate that these two lipases have distinct physiological effects. **This is an important finding that has implications in our understanding of mechanisms that underlie innate immunity, fat metabolism, reproductive fitness, and how these processes are coordinated. The data are rigorous and clearly presented, and this work will be of interest across fields.**

I think that this work would be even stronger if the authors could more clearly show that the changes they observe were functionally relevant. A couple of examples:

* It is not clear if observed changes in embryo permeability are related to the fertility defect; whereas increased permeability is only observed in the tcer-1;lipl-2 double mutant, embryo viability, sterility, or brood size are all similarly reduced by lipl-1 and lipl-2. Moreover, the fraction of embryos permeable to dye (1.5% according to Fig S4) is much lower than the fraction of embryos that don't hatch shown in Fig 5C. While it is certainly interesting that lipl-2 has this effect on permeability, It seems like there could be other tcer-1-associated changes that may be more germane to the effect on fertility observed. How do the authors explain the lack of correlation between permeability and the fertility defect they observe? Is it possible that the reduced brood size (Fig 5A) is reflecting a defect in sperm production or function? Does PA14 exposure exacerbate the permeability effect?

We appreciate the reviewer raising these thoughtful and important questions. We agree that the fertility and hatching defects observed in our study cannot be solely attributed to increased embryonic permeability alone. Indeed, the proportion of embryos exhibiting porous eggshells is smaller than the overall fraction that fail to hatch. Nonetheless, eggshell disruption is invariably fatal and thus represents one significant contributor to reduced reproductive success.

Previous work from our group (Amrit et al., *PLoS Genetics*, 2016; PMID: 26862916) has demonstrated that *tcer-1* mutants exhibit intrinsic defects in both sperm and oocyte function. In addition, in an independent, unpublished study, we have found that *tcer-1* mutant mothers show

impaired deposition of specific fatty acids and lipid species into embryos during pathogenic infection. This is a transport defect linked to *tcer-1*- dependent regulation of branched-chain fatty acid metabolism wherein the porosity defects are exacerbated by infection. It is the focus of another article under preparation.

Taken together, our findings suggest that the fertility phenotypes associated with *tcer-1* inactivation, particularly upon pathogenic infection, arise from multiple intersecting defects including compromised lipid mobilization mediated by LIPL-1 and LIPL-2. While it remains challenging to disentangle the relative contributions of each defect, our current data clearly identify these two lysosomal lipases as important components of the broader network through which TCER-1 regulates reproductive fitness.

* The authors show that there are some changes in the lipid profile of tcer-1 are related to lipl-1 and/or lipl-2, but there is no data to indicate which, if any, of these changes are functionally relevant. Neither lipl-1 nor lipl-2 impact the DAG or TAG of tcer-1 mutants (Fig A,B), suggesting that these are not relevant to the phenotypes of the double mutants. In contrast, there are some tcer-1-associated changes in lipids that do seem to be differently impacted by lipl-1 and lipl-2 (such as ceramides Cer17:1; O2/22:0 and Cer17:1; O2/24:0). It would be interesting to know if any of the tcer-1 immunity or fertility phenotypes are sensitive to orthogonal perturbations in these lipids. It would also be interesting to know how the authors are thinking about the pattern of lipid changes - why do some ceramides change, but not others? Why are some unsaturated lipids more affected than others?

We appreciate the reviewer's insightful questions. As mentioned above in response to Reviewer #1's Point 3 (and shown in Fig.5J, K) we demonstrate that at least one of the specific lipid species whose levels are impacted by TCER-1 in a LIPL-1-dependent manner- the ceramide, Cer 24- is functionally important. Cer 24 supplementation rescues the survival of *tcer-1;lipl-1* mutants and improves the survival of WT animals as well.

A particularly revealing aspect of our study is the alteration of specific, functionally relevant sphingolipid species in a lipase-dependent manner. LIPL-1 and LIPL-2 did not broadly alter global neutral lipid or phospholipid levels but instead modulated select species, notably ceramide sphingolipids. This aligns with growing evidence that overall fat or TAG levels are poor indicators of lifespan or stress resilience: long-lived insulin/IGF-1 mutants such as *daf-2* and germline-ablated animals accumulate fat, whereas calorie-restricted animals are lean yet long-lived (PMIDs: 19883620, 23935515).

Ceramides are essential membrane lipids with critical signaling roles, and distinct species in *C. elegans* have been linked to mitochondrial surveillance (Cer 24), heat-stress resistance (Cer 16), and germline ablation- induced longevity (GlcCer C22) (PMIDs: 36787434, 19372430, 40461452). The specificity of such ceramide changes likely reflects the influence of their acyl chain composition, which affects lipid shape, protein interactions, and function. We do not yet understand why these specific ceramide species or unsaturated fatty acids are impacted and others are not, but it is likely reflective of the substrates LIPL-1/-2 act on and the local cellular environment the enzymes operate in. Pertinently, while alterations in ceramides associated with stress and longevity have previously been attributed to regulation of biosynthetic enzymes such as the ceramide synthase *hyl-2* (PMID: 36787434) our RNA-seq data did not show the

differential expression of *hyl-2* or other sphingolipid biogenesis genes following pathogen infection or *tcer-1* loss. Hence, these observed immune-related ceramide changes appear lipolysis-driven, consistent with ceramide generation by lysosomal lipases through breakdown of complex sphingolipids.

Our data with Cer 24 supplementation underscore the physiological significance of such specific lipid species. Analogously, in humans, distinct ceramides rather than total lipid content determine metabolic outcomes. For instance, elevated C16:0 and C18:0 ceramides correlate with insulin resistance and β-cell dysfunction in Type 2 Diabetes (PMID**:** 19008343**,** 25295788), whereas, exercise selectively reduces C16:0 and enriches very-long-chain ceramides (C24:0, C24:1), enhancing insulin sensitivity and mitochondrial capacity (PMID: 25966363). Together, these findings highlight that targeted remodeling of ceramide composition, not total fat content, underlies adaptive and pathological metabolic states.

We have revised the Discussion section to include these points (Pgs 20, 21; Lines 385-413).

I would also like to raise some **minor points** that I hope the authors could address that I believe would improve the manuscript:

* It's not entirely clear to me how the authors identified the lipl genes from the RNAseq data presented. The way the manuscript is presented, it seemed that the authors were most interested in tcer-1-dependent, PA14 responsive transcriptional changes but it is a bit confusing which set of differentially expressed genes (DEG) they include in this set. The text suggests that the genes of interest would be included in the WT/PA14 vs tcer-1/PA14. However, some of these genes (including lipl-1 and lipl-2) are also included in the tcer-1/OP50 vs tcer-1/PA14 DEG, which the authors indicate are "tcer-1 independent" transcriptional responses. It could also be argued that the DEG in the wild-type PA14 response (WT/OP50 vs WT/PA14) that do not change in tcer-1 (OP50 vs PA14) are also "tcer-1-independent" responsive.

We focused on *lipl-1* and *lipl-2* because PA14 infection and TCER-1 appeared to exert ***opposite*** effects on their expression. In WT animals, both genes were upregulated upon infection, but, in *tcer-1* mutants, this upregulation was further amplified (Fig. 1H, I). This was in contrast to the impact on *lipl-3, lipl-4* and *lipl-5* (data now included as Fig. 1J-L) whose expressions were reduced in *tcer-1* mutants and upon PA14 infection. We found the *lipl-1/lipl-2* data intriguing because it suggested that, in WT animals, *lipl-1* and *lipl-2* may be induced as part of the immune response but repressed when TCER-1 is active; in *tcer-1* mutants, the absence of this repression could lead to an elevated immune activation. These opposing effects were consistent with (and to us, indicative of) a potential role for *lipl-1* and *lipl-2* in the TCER-1- mediated tradeoff between immunity and fertility. This reasoning motivated our focus on the genes for this study. The rationale has been elaborated in the Results section (Pgs. 9, 10; Lines 145-165).

We fully agree with the reviewer that TCER-1- dependent and -independent targets can be defined in multiple ways. Some of the confusion likely arose from an inadvertent mislabeling of two DEG lists: the lists of genes upregulated and downregulated in *tcer-1*/PA14 vs. WT/PA14 were mistakenly reversed (Table S1Di and Dii). We apologize for this error, which has now been corrected. To enhance clarity, we have revised the manuscript to indicate the condition and

directionality of gene-expression changes without attributing specific dependency relationships to them (Pg. 8; Lines 121-129).

* I don't understand why the authors didn't use the DEG in PA14 that were tcer-1 dependent (WT/PA14 vs tcer-1/PA14) in the wormcat analysis shown in Fig 1G. The way these data are presented, there does not seem to be much difference in the WT and tcer-1 responses. It is really not clear how this result helped to identify the lipl genes.

We thank the reviewer for this helpful comment and apologize for any confusion regarding our selection of the *lipl* genes for this study. As mentioned above, the *lipl* genes drew our attention because *tcer-1* mutation and PA14 infection had opposite effects on their expression. While we conducted WormCat analyses in multiple ways, including comparisons between WT/PA14 and *tcer-1*/PA14 datasets, we chose to highlight in the main figure the analysis that most clearly illustrated the reciprocal effects of PA14 infection and *tcer-1* loss. For example, in Fig. 1G, the 'Metabolism' category is enriched (Category 1) in both WT and *tcer-1* mutants within the PA14 vs. OP50 UP class, but the enrichment is stronger and more significant in *tcer-1* mutants-mirroring the mutants' heightened induction of *lipl-1* and *lipl-2*. We have revised the manuscript to clarify our rationale for focusing on *lipl-1* and *lipl-2* (Pgs. 9, 10; Lines 145-165) and have included additional WormCat analyses in Supplementary Figure 2.

* Do any of the other 6 lipl genes change in response to PA14?

Yes. Among the '*lipl'* gene family, two distinct subsets emerged based on the opposing effects of PA14 infection and TCER-1 on their expression. While *lipl-1* and *lipl-2* were upregulated upon PA14 exposure, *lipl-3*, *lipl-4*, and *lipl-5* were downregulated. This contrasting regulation within the same gene family was one of the reasons we focused on the *lipl* genes. The data for *lipl-3*, *lipl-4* and *lipl-5* have now been included in the revised manuscript (Fig. 1J-L).

* It seems that in Fig 1I the authors statistically compare lipl-2 expression between WT/PA14 and tcer-1/OP50. It is not clear why this comparison would be considered instead of analyzing these data the same as for lipl-1 in Fig 1H.

We have edited the figure to show the statistical significance between WT/PA14 vs. *tcer-1*/PA14 as well as *tcer-1*/OP50 vs *tcer-1*/PA14 (both were highly significant).

* The authors compare these RNAseq data to previous published transcriptomic analysis of PA14 responses. While there are significant overlaps in the genes that change in each data set, as expected, there are a lot of changes that do not overlap. Do the authors have an idea for why these data sets are so different?

We believe the incomplete overlap among datasets is expected given the substantial differences in experimental designs, sequencing platforms, and analytical pipelines across studies. For example, earlier investigations (*Troemel et al., 2006; Shapira et al., 2006*) employed microarrays, whereas our analysis used RNA-seq, which provides greater dynamic range and sensitivity. Indeed, the more striking observation is not the divergence but rather the extent of concordance despite these methodological differences. Even within a single study, *Troemel et al. (2006)* found only partial overlap between genes induced after 4 and 8 hours of PA14 exposure in WT animals- of 310 and 269 upregulated genes after 4h and 8h, respectively, only 197 were shared (63.5% of the 8-hour DEGs). By comparison, the substantial overlaps we

observed between our dataset and studies spanning nearly two decades underscore not only the reproducibility of previously reported infection-responsive genes but also the robustness of the newly identified candidates in our study.

* The authors suggest that the LIPL reporters are expressed in the head of a small fraction of worms. I'm not convinced that this reflects the normal expression pattern of these genes, and I think including this observation in the main figures is somewhat misleading. Unless the authors want to do more experiments with relevant controls, I would recommend that they do not include pictures of these rare animals in the figures. Similarly, the author claim that this low and uncommon expression is in neurons but it seems that it might also be hypodermis or pharynx. This should be corrected unless the authors can show colocalization with a neuronal marker.

We thank the reviewer for this suggestion and agree with them. While we are confident of the quantification of the transcriptional reporters, the expression in the translational reporters is indeed highly variable in the head/neurons (the coelomocyte and intestinal expression are low but consistent). We have removed the images of the protein expression in the head from the manuscript. We have moved the images of intestinal protein expression to a supplementary figure (Fig. S5) as we believe these will be helpful to the readers in assessing overall expression levels/pattern. The manuscript has also been edited to indicate this. In the revised version, Fig. 2 shows the expression of the transcriptional reporters of both genes as well as the quantification under different conditions. The details of the quantification protocol as well as diversity of expression within the population is shown in Fig. S3 (*lipl-1*) and Fig. S5 (*lipl-2*) and described in the Methods section (Pgs. 26, 27; Lines 521-527).

* The authors show images of a single worm to support their claims in Fig 2, SFig 2, Fig 3, and Sfig 3. Especially when there is not a quantitation of a population, showing a single worm is not sufficient of the reader to evaluate if these data support the stated conclusions.

As mentioned above, the images of transcriptional reporters in Fig. 2 are representative of the results obtained after detailed quantifications, which are shown in the figure as well. Further, the supplementary figures show examples of the high, medium and low categories of worms as well as the 'occurrence' of these categories in different conditions/ backgrounds/populations. This is shown for both *lipl-1* (Fig. S3) and *lipl-2* (Fig. S5).

We agree with the reviewer's assessment about limitations of single worm images. We were unable to quantify the impacts of *tcer-1* inactivation and/or PA14 infection on the translational reporters though are confident of the trends we observed, hence have moved the translational-reporter images to supplement (Fig. S4).

* It is not clear how many independent replicates were done for lifespan experiments, or if the Figure shows a single experiment or if it is an aggregation of all replicates. This should be made clear in the figure legend, and summary statistics for all replicates should be included in the Supplementary Materials (mean survival, max survival, # animals, # censored, etc).

Three independent trials of lifespan experiments were conducted. The results including mean survival, censoring details and statistics are included in Table S4. For the data shown in Fig. 3B and D (previously Fig. 4), the survival data and statistics are included in the figure legend (Pg. 37).

* In Fig 5, the authors claim to measure the "hatch rate", but this is not a rate measurement, it is just the fraction of the eggs that hatch. It is also not clear how the authors defined "sterility" for the data shown in Fig 5B.

We have replaced 'hatch rate' with 'hatching' throughout the manuscript. Sterility was measured in a reproductive-health assay by counting animals that failed to lay any viable embryos and this fraction used to estimate sterility in the population. This definition has been added to the Methods section that includes further details of the reproductive-health assays (Pg. 29; Lines 582-583).

* It is not clear how many replicates are included in the lipidomics experiments and this makes it difficult to interpret the data. Many of the changes seem pretty small, and it would be useful to know how the abundance varies across samples to have a better understanding of the robustness of the changes that are noted.

Overall, worms grown in five or six independent biological replicates were collected to perform the lipidomics. Some strains (*tcer-1, tcer-1;lipl-1* and *tcer-1;lipl-2*) showed asynchronous and/or delayed development as well as varying infertility. To ensure that only age-matched, fertile young adults were included in the analyses, individuals with eggs were manually collected for these strains. Due to the labor-intensive nature of this approach, the sample collection was performed in two batches with WT and *tcer-1* controls included in each batch: (i) WT, *lipl-1*, *tcer-1*, *tcer-1;lipl-1* and (ii) WT, *lipl-2*, *tcer-1*, *tcer-1;lipl-2.* Following lipid extraction and separation into neutral lipid, phospholipid and sphingolipid fractions, 7-10 successful MS runs of WT and *tcer-1* samples and 4-6 successful runs of the other strains were accomplished and these data was included in the lipidomic analyses. These details have been added to the Methods section (Pg. 31, Lines 629-635; Pg. 33, Lines 664-666).

* Is the hLAL protein also localized to the coelomocytes? Other work suggests that localization to coelomocytes is important for LPL function (Buis et al 2019) – is this true for tcer-1 associated effects of lipl-1 and/or lipl-2?

Our preliminary examination of the hLAL/LIPA strains did not detect localization to a specific organelle, possibly in part because of the high level of fluorescence. Assessing the subcellular localization of the worm LIPLs and the hLAL/LIPA protein is the focus of our ongoing/future studies.

* On page 20 it would be good to include the relevant reference where the plipl-2::LIPL-2::RFP transgenic strain was first characterized.

We apologize for the omission. The reference has been added.

* There are some unformatted citations on pg 24, 28, and 29.

The references have been corrected.

* In the materials and methods the authors should replace "u" with the greek mu where Appropriate

The corrections have been made.

* For pathogen exposure experiments, how many independent replicates were performed? Is the data shown in the figures aggregated data or a representative experiment? The authors should include this information in the legends, and also provide summary statistics for independent replicates in the supplementary material. Also, the authors mention S. aureus in the methods section but I don't think any of these experiments is included in this manuscript.

All survival assays were conducted in at least two independent trials, often 3-5 trials. The figures show data from a single trial and the corresponding legend lists the survival and summary statistics. Data from all trials are shown in the corresponding supplementary table indicated in the figure legend. For instance, the data shown in Fig. 3 (previously Fig. 4) for survival on PA14 and OP50 is summarized in the associated legend, and data from all trials shown in Tables S3 and S4, respectively (Pg. 37). As suggested, the data on *S. aureus* survival assays has been removed from the manuscript.

* The authors used FuDR to prevent egg hatching on PA14, but don't show controls to make sure that FuDR doesn't affect the results of these assays. It would be nice to see this control.

We have compared the survival of WT animals and *tcer-1* mutants upon PA14 infection in the presence and absence of FUDR and found comparable results. One example is shown here and additional data included in the manuscript as Table S7B.

**Table: Impact of FUDR on PA14 Response**					
**Treatment**	**Strain**	**Obs/Total**	**Mean (Hrs)**	**SEM**	**P vs WT**
		**Trial 1**			
FUDR	WT	124/128	66.07	1.02	
	tcer-1	106/122	80.01	1.67	<0.0001
		**Trial 2**			
NO FUDR	WT	64/153	62.22	1.61	
	tcer-1	115/152	79.84	2.06	1.30E-08

* For experiments comparing transgene expression, what calibrations were performed to ensure that comparisons between samples are valid? Also, were the samples blinded in any way before the assay?

For each transgenic strains generated/reported in this study, we confirmed that at least two (often more) independent 'lines' showed similar expression patterns before selecting one line for detailed studies. The basal expression in the population was characterized extensively to determine the sorting of the population into 'high', 'medium' and 'low' expression individuals, as shown in Supplementary Figures S3 (*lipl-1*) and S5 (*lipl-2*) and described in Methods. For experiments comparing strains/conditions, investigators were blinded as much as possible (for eg., OP50 vs. PA14 plates look obviously different from each other, but the experiment was done in a darkened room to reduce bias). Lastly, each experiment was repeated at least three independent times for reproducibility.

* In the materials and methods the authors suggest that they separated young adults from other stages for lipidomics experiments with strains that were not easily synchronized. Was this done manually? It is not clear what exactly was done to achieve this.

Yes, this was performed manually. This has been detailed/clarified in the Methods section (Pg.31; Lines 629-653).

Reviewer #3

Using the C. elegans model, Bahr et al. investigate the role of two lipases, LIPL-1 and -2 in the regulation of life span, immune responses, and reproduction, both in wild type worms in the tcer-1 mutant background. Deploying lipidomic, trascriptomic, and genetic (mutant/rescue and reporter) approaches they find that these two lipases are regulated substantially in tcer-1 mutants and after infection with the Pseudomonas aerigunosa strain PA14. Despite the similar regulation of both genes, their loss of function causes disparate effects, with lipl-1 required for the enhanced resistance of tcer-1 to pathogen, and tcer -1 for the life span phenotype. Both genes showed defects, and synthetic effects on various parameters of reproduction. Excitingly, lipidomics revealed some distinct changes between lipl-1 and -2, for example the blocked induction of 3 ceramide species by lipl-1 mutation in the tcer-1 context. Finally, the authors showed that the human LIPL ortholog, hLAL, could rescue the defective pathogen resistance caused by lipl-1 mutation.

This study provides important new insight into both the phenotypes and underlying molecular lipid defects of two lipases and should be of interest to researchers studying gene regulation, metabolism, immune responses, and reproduction. The experiments are overall well done and very comprehensive.

We thank the reviewer for their thoughtful and generous assessment of this work. We appreciate their recognition that our study provides new insights into the phenotypic and molecular consequences of lipase disruption, with broad relevance to gene regulation, metabolism, immunity, and reproduction, as well as their acknowledgment of the rigor and scope of our experiments.

Comments:
- It would be exciting to be able to show whether the deregulated ceramide species can be supplemented to rescue the defects in immune defence; I appreciate that this may be a challenging experiment however.

We thank the reviewer for this excellent suggestion. As described and shown above, in response to Reviewer #1's comment 3, supplementation with Cer 24 (the species elevated in *tcer-1* mutants dependent upon *lipl-1*) rescues the pathogen resistance of *tcer-1;lipl-1* mutants and enhances the post-infection survival of WT animals as well. These data are now included in the manuscript (Fig. 5J, K and Table S7).

- I was surprised that the authors didn't also attempt to test whether hLAL also rescues lipl-2 mutant phenotypes

We have included the results of our tests regarding hLAL's rescue of *tcer-1;lipl-2* (and *tcer-1;lipl-1*) fertility phenotypes in the revised manuscript. Overall, our experiments provided equivocal data; there was a trend towards rescue of the brood size and the embryonic-permeability phenotypes of *tcer-1;lipl-2* mutants but there was significant variability between strains and trials and the rescue did not achieve statistical significance. These data are depicted in Fig. 6C-E and shown above in response to Reviewer #1's comment 4.

- Results P13: conclusion "lipl-2 single mutants also showed a similar change in TAG:DAG distribution which was further aggravated in tcer-1;lipl-2 double mutants." Differences between the double and the singles appear minor and not stats are shown - did the authors mean to write distribution was NOT further aggravated? I would reason that that is what's supported by the data and statistics.

The reviewer is correct in pointing out the lack of statistical significance of this observation although there was a clear trend of further enhancement of the ratio in *tcer-1;lipl-2* double

mutants as compared to either single mutant. We have amended the statement to point out that the increase was small and statistically not significant (Pg. 15; Lines 288-290).

Typos
- Abstract: "lipl-l"
- Intro p4: "Arabidopsis" add "thaliana"
- Results p9: "LIPL-1::RFP expression showed similar spatial distribution as the mRNA" – GFP reporters measure promoter activity, not mRNA. Rephrase.
- Results p12: "the lipl-1 or lipl-1 mutations"
- Results p15 "LPC 18: 2" delete space
- P18 discussion: "lOur"

We apologize for the typos and have corrected these.

Results p12 "even reproductively older Day 4" sounds odd - aren't they simply "older"?

We used the qualifier 'reproductively older' to clarify that, while Day 4 animals are relatively young in terms of total lifespan (>2 weeks), they are considered old with respect to their reproductive age.
